# Stabilization of Microtubule-Unbound Tau via Tau Phosphorylation at Ser262/356 by Par-1/MARK Contributes to Augmentation of AD-Related Phosphorylation and Aβ42-Induced Tau Toxicity

**DOI:** 10.1371/journal.pgen.1005917

**Published:** 2016-03-29

**Authors:** Kanae Ando, Akiko Maruko-Otake, Yosuke Ohtake, Motoki Hayashishita, Michiko Sekiya, Koichi M. Iijima

**Affiliations:** 1 Department of Biological Sciences, Graduate School of Science and Engineering, Tokyo Metropolitan University, Hachioji, Tokyo, Japan; 2 Department of Neuroscience, Thomas Jefferson University, Philadelphia, Pennsylvania, United States of America; 3 Department of Alzheimer’s Disease Research, National Center for Geriatrics and Gerontology, Obu, Aichi, Japan; Stanford University School of Medicine, UNITED STATES

## Abstract

Abnormal accumulation of the microtubule-interacting protein tau is associated with neurodegenerative diseases including Alzheimer’s disease (AD). β-amyloid (Aβ) lies upstream of abnormal tau behavior, including detachment from microtubules, phosphorylation at several disease-specific sites, and self-aggregation into toxic tau species in AD brains. To prevent the cascade of events leading to neurodegeneration in AD, it is essential to elucidate the mechanisms underlying the initial events of tau mismetabolism. Currently, however, these mechanisms remain unclear. In this study, using transgenic *Drosophila* co-expressing human tau and Aβ, we found that tau phosphorylation at AD-related Ser262/356 stabilized microtubule-unbound tau in the early phase of tau mismetabolism, leading to neurodegeneration. Aβ increased the level of tau detached from microtubules, independent of the phosphorylation status at GSK3-targeted SP/TP sites. Such mislocalized tau proteins, especially the less phosphorylated species, were stabilized by phosphorylation at Ser262/356 via PAR-1/MARK. Levels of Ser262 phosphorylation were increased by Aβ42, and blocking this stabilization of tau suppressed Aβ42-mediated augmentation of tau toxicity and an increase in the levels of tau phosphorylation at the SP/TP site Thr231, suggesting that this process may be involved in AD pathogenesis. In contrast to PAR-1/MARK, blocking tau phosphorylation at SP/TP sites by knockdown of Sgg/GSK3 did not reduce tau levels, suppress tau mislocalization to the cytosol, or diminish Aβ-mediated augmentation of tau toxicity. These results suggest that stabilization of microtubule-unbound tau by phosphorylation at Ser262/356 via the PAR-1/MARK may act in the initial steps of tau mismetabolism in AD pathogenesis, and that such tau species may represent a potential therapeutic target for AD.

## Author Summary

Alzheimer’s disease (AD) is the most common cause of dementia resulting from progressive neuron loss. Two proteins, β-amyloid (Aβ) and tau, accumulate in AD brains and are involved in AD pathogenesis. In healthy neurons, tau binds to microtubules to regulate its stability; in AD brains, however, tau is detached from microtubules and phosphorylated at multiple sites. Such abnormal tau behavior, which is likely to be triggered by Aβ, results in generation of pathological tau species that mediate neuron loss. However, the detailed mechanisms underlying this event remain incompletely understood. Using transgenic flies expressing human tau and Aβ as a model system, we found that tau phosphorylation at specific AD-related sites stabilized microtubule-unbound tau in the early phase of tau mismetabolism to generate toxic tau species. Moreover, this process is critical for Aβ to promote subsequent tau phosphorylation and neurodegeneration. Our results reveal a critical step in the initiation of tau mismetabolism, and this process may represent a potential therapeutic target for AD.

## Introduction

Alzheimer’s disease (AD) is a progressive neurodegenerative disease characterized by two pathological lesions: deposition of β-amyloid peptides (Aβ) as amyloid plaques and the microtubule-associated protein tau in the form of paired helical filaments in neurofibrillary tangles (NFTs) [1]. Genetic, pathological, and biochemical evidence suggests that elevation of Aβ levels is a causal event in AD pathogenesis [2–8] that lies upstream of tau-induced neurodegeneration [3, 5, 9, 10]. In AD and other neurodegenerative diseases, collectively referred to as tauopathies, tau protein self-aggregates into multiple intermediate forms, including soluble oligomers and prefibrils, that may ultimately form insoluble NFTs [11]. These tau aggregates all exert neurotoxicity, with some qualitative and quantitative differences; the soluble, prefibrillar aggregates are thought to cause the most damage to neurons [12–14]. To prevent the cascade of events leading to neurodegeneration in AD, it is crucial to elucidate the mechanisms underlying the initial steps of abnormal metabolism of tau.

Tau proteins are normally enriched in neuronal axons, where they regulate microtubule stability. However, in diseased brains, tau is detached from microtubules and aggregated in the cytosol. The microtubule-binding domain of tau mediates interaction to proteins, including tau itself, which can cause self-aggregation into oligomers, protofibrils, and fibrils [15–20]. In addition, tau is abnormally phosphorylated in diseased brains [21–24], and tau proteins detached from microtubules are prone to be phosphorylated at disease-associated sites [19, 25–28]. Moreover, tau detached from microtubules can mislocalize to dendrites and extracellular regions, where it can disrupt neuronal functions or spread into other neurons [29–34]. These observations suggest that the loss of tau binding to microtubules may be a triggering event for abnormal metabolism of tau. However, the detailed molecular mechanisms underlying this event and how it relates to Aβ-mediated tau toxicity remain elusive.

Tau is phosphorylated at more than 40 sites in pathological lesions associated with AD [21–24], and Aβ promotes tau phosphorylation at disease-associated sites in *in vitro* and *in vivo* models of AD [2, 35–41]. A number of kinases and phosphatases regulate the phosphorylation status of tau [42], and the activities of two major tau kinases, GSK3 and PAR-1/microtubule affinity-regulating kinases (MARKs), are often associated with tau detachment from microtubules and Aβ-induced augmentation of tau toxicity [37, 39, 43–45]. GSK3 is a proline-directed kinase that contributes to phosphorylation of tau at serine or threonine followed by proline (i.e., SP/TP sites) [46–49], whereas PAR-1/MARKs are non-SP/TP kinases that phosphorylate tau at Ser262 and Ser356 in the repeat domains located in the microtubule-binding region [50]. Tau phosphorylation at these sites decreases the protein’s affinity toward microtubules *in vitro*, in cultured cells, transgenic *Drosophila*, and transgenic mice [18, 19, 25–27, 50–52].

Tau phosphorylation at GSK3-target sites, as well as Ser262/356, has prominent effects on tau toxicity [26, 53–56]. In cellular and animal models, co-expression of Aβ augments tau phosphorylation at these sites and increases the levels of active GSK3 and PAR-1/MARK [2, 35–41, 43, 44]. Notably, studies in cultured neurons and transgenic *Drosophila* showed that Aβ-induced augmentation of tau toxicity caused by Aβ depends on tau phosphorylation at these sites [39, 45, 56–58]. These observations demonstrate the importance of tau phosphorylation by GSK3 and MARK in the abnormal metabolism and toxicity of tau. However, the roles of, and interdependency between, tau phosphorylation by GSK3 and MARK/PAR-1 in an early step of tau mismetabolism, such as tau mislocalization and stabilization of mislocalized tau, are not fully understood.

*Drosophila* models expressing human tau recapitulate key features of human tauopathies, including progressive neurodegeneration and phosphorylation at AD-related sites via conserved kinases [41, 44, 53, 59–63]. Formation of fibrils or detergent-insoluble aggregates are not detected in the presence or absence of Aβ [58, 59], suggesting that the neurodegeneration observed in these models reflects toxicity of soluble, non-aggregated forms of tau, and may recapitulate early stages of the abnormal metabolism and toxicity of tau [11–14].

In this study, we investigated the roles of tau phosphorylation at Ser262/356 by PAR-1/MARK and SP/TP sites by GSK3 in an initial step of tau mismetabolism, using transgenic *Drosophila* co-expressing human tau and Aβ42 in neurons [59, 64]. We found that augmentation of tau toxicity by Aβ42 was concomitant with increased levels of microtubule-unbound free tau in the cytosol, regardless of its phosphorylation status. Tau phosphorylation at Ser262/356 by PAR-1/MARK preferentially stabilized less phosphorylated forms of microtubule-detached tau in the cytosol, and blocking this stabilization of tau suppressed Aβ42-induced augmentation of tau toxicity and the increase in the levels of tau phosphorylated at the AD-associated residue Thr231. By contrast, blocking tau phosphorylation at GSK3-target SP/TP sites did not affect tau stability or suppress the mislocalization and toxicity of tau induced by Aβ42. These results suggest that detachment of tau from microtubules, followed by stabilization by phosphorylation at Ser262/356 via PAR-1/MARK, is a critical early step in the formation of tau species associated with neurodegeneration, and that tau mismetabolism via this pathway is facilitated by Aβ42.

## Results

### Aβ42 increases the level of tau in the cytosol and decreases the level of microtubule-bound tau

**Fig 1:**
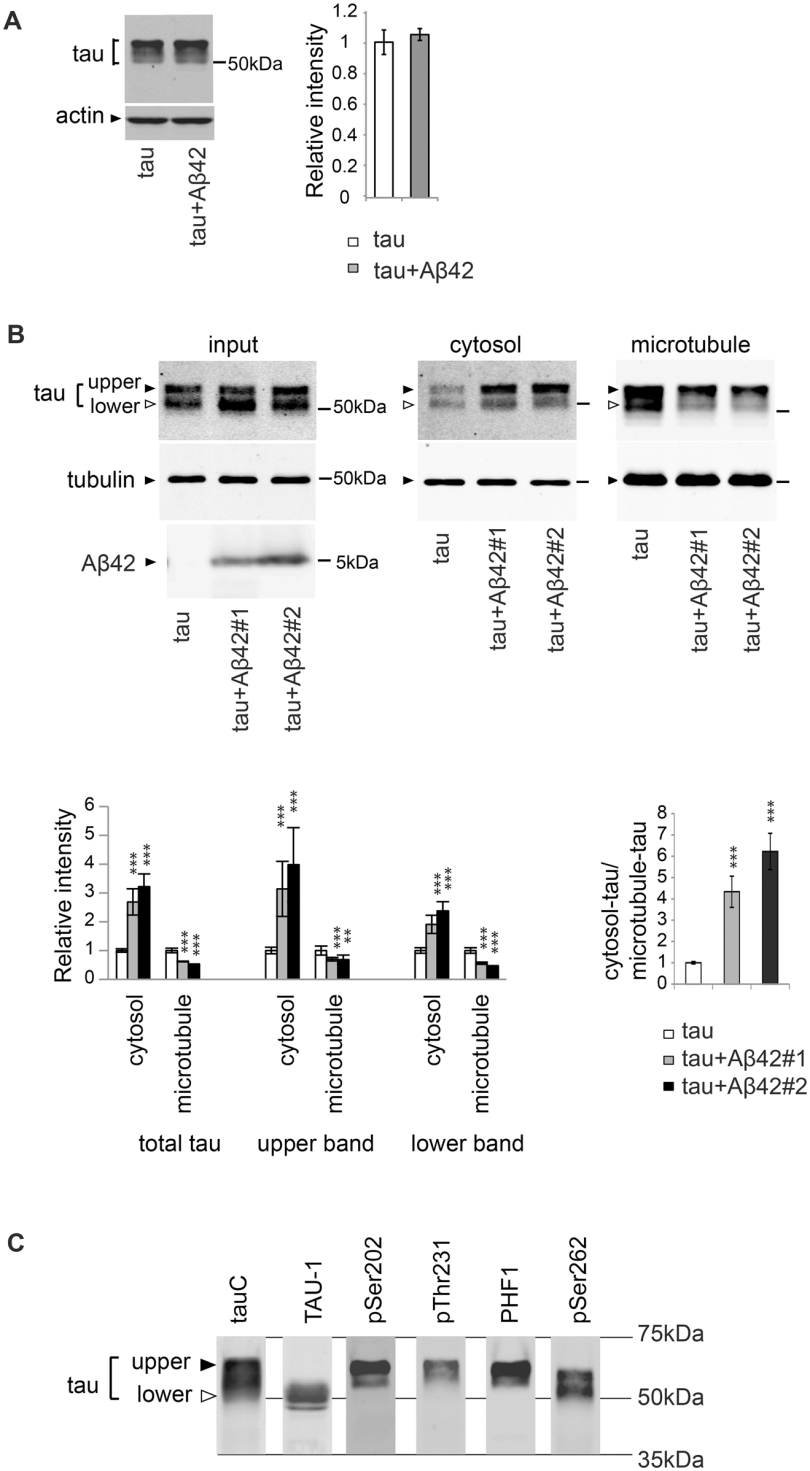
Aβ42 increases the level of tau in the cytosol and decreases the level of microtubule-bound tau. (A) Aβ42 does not change total tau levels. Western blot of heads of flies expressing tau alone (tau) or that co-expressing tau and Aβ42 (tau+Aβ42) driven by gmr-GAL4 with anti-tau antibody. Actin was used as a loading control. Mean ± SD, n = 5; no significant difference was found by Student's t-test (*p*>0.05). Representative blots are shown. (B) Co-expression of Aβ42 increases the levels of tau free from microtubules and reduces the levels of tau bound to microtubules. The levels of tau and tubulin in the lysate of fly heads expressing tau alone (tau) or co-expressing tau and Aβ42 (tau+Aβ42#1 and tau+Aβ42#2) before sedimentation (input), in the supernatant (cytosol) and in the pellet containing microtubules (microtubule) were analyzed by western blotting by using anti-tau antibody. The same amount of proteins from each genotype was loaded. Expression of Aβ42 was confirmed by western blot with anti-Aβ antibody (Aβ42). Two independent transgenic fly lines expressing Aβ42 at different expression levels (Aβ42#1 and Aβ42#2) yielded similar results, and the fly line with higher Aβ42 (Aβ42#2) expression exhibited a larger effect. Transgene expression was driven by gmr-GAL4. Mean ± SD, n = 4; **, *p*<0.01, ***, *p*<0.005 compared to tau by one-way ANOVA with Tukey's post hoc test. Representative blots are shown. (C) Two major bands detected by western blotting of fly heads expressing tau with a pan-tau antibody (tauC) differ in their phosphorylation patterns. Western blots with TAU-1 antibody, which specifically recognizes tau protein without phosphorylation at several AD-related sites (Ser194, Ser195, Ser198, and Ser202) (TAU-1), anti-phospho-Ser202, anti-phospho-Thr231, anti-phospho-Ser396/404 (PHF1), or anti-phospho-Ser262 antibody (pSer262) are shown. Transgene expression was driven by gmr-GAL4.

To understand the mechanisms by which abnormal metabolism and toxicity of tau is triggered by Aβ42, we analyzed changes in human tau induced by co-expression of human Aβ42 in transgenic flies [58]. Because accumulation of tau is a hallmark of AD brains, and an increase in tau levels positively correlates with tau toxicity in cellular and animal models [65], we first investigated whether tau accumulation occurred in association with Aβ42-induced augmentation of tau toxicity. As reported previously, co-expression of Aβ42 in fly eyes using the pan-retinal driver gmr-Gal4 enhanced tau-mediated retinal degeneration ([58] and S1 Fig). In this model, the levels of tau did not differ significantly between flies expressing tau alone and those expressing tau and Aβ42 (Fig 1A), suggesting that Aβ42-mediated augmentation of tau toxicity is not simply due to an overall increase in the level of tau [58].

Most tau proteins are normally bound to microtubules in axons, and alterations in the distribution of tau are associated with tau toxicity [66]. To determine whether mislocalization of tau occurred in our fly models, we analyzed the effect of Aβ42 expression on tau binding to microtubules using an *in vivo* microtubule-binding assay [25]. In this assay, endogenous microtubules and microtubule-bound proteins were present in the pellet following centrifugation (microtubule fraction), whereas cytosolic proteins free from microtubules were recovered in the supernatant (cytosol fraction). The levels of tau in each fraction were determined by western blotting with anti-tau antibody. We found that the levels of tau in the cytosol fraction were significantly elevated, whereas the levels in the microtubule fraction were reduced, in the fly retina co-expressing Aβ42 and tau (Fig 1B). These experiments were carried out using two independent transgenic fly lines expressing Aβ42 at different expression levels. Both lines yielded similar results, and the fly line with higher Aβ42 expression exhibited a larger effect (Fig 1B, compare Aβ42#1 and Aβ42#2). We also investigated whether co-expression of non-toxic proteins in the secretory pathway (CD8-GFP) altered tau distribution non-specifically. Expression of CD8-GFP did not change the levels of tau in either the cytosol fraction or the microtubule fraction (S2 Fig), suggesting that alterations in tau distribution are associated with Aβ42-mediated toxicity.

Tau proteins expressed in the fly retina are detected as two major bands by western blotting with pan-tau antibody (Fig 1B). Following phosphatase treatment, these two bands merged and were detected as a single faster-migrating band (S3 Fig). This observation indicates that the differences between these two tau bands are related to their phosphorylation levels: tau in the slower-migrating band (tau^upper^) is more highly phosphorylated than tau in the faster-migrating band (tau_lower_).

To further characterize phosphorylation profiles of tau^upper^ and tau_lower_, we used a panel of antibodies capable of distinguishing the phosphorylation status of tau at AD-related sites. Western blotting with TAU-1 antibody, which specifically recognizes tau protein without phosphorylation at several AD-related sites (Ser194, Ser195, Ser198, and Ser202), detected tau_lower_ (Fig 1C, TAU-1), whereas antibodies specific to phospho-Ser202, phospho-Thr231, or phospho-Ser396/404 (PHF1) preferentially recognized tau^upper^ (Fig 1C, pSer202, pThr231, and PHF1, respectively). By contrast, phospho-Ser262-specific antibody recognized both tau^upper^ and tau_lower_ species (Fig 1C, pSer262).

In the Aβ42 fly, levels of both tau^upper^ and tau_lower_ were elevated in the cytosol fraction (Fig 1B, cytosol), but reduced in the microtubule fraction (Fig 1B, microtubule). These results suggest that, regardless of its phosphorylation status, microtubule-unbound free tau in the cytosol is more abundant in the presence of Aβ42.

Cytoskeletal pathologies, including microtubule disintegrity, have been reported in AD brains and cultured cells incubated with Aβ [39, 67–69]. Under this particular experimental condition, however, co-expression of Aβ42 did not significantly change the levels of tubulin in the form of microtubules (Fig 1B, tubulin) or the levels of acetylated or tyrosinated tubulin (S4 Fig), suggesting that the stability of microtubules was not grossly affected by Aβ42.

### Tau phosphorylation at SP/TP sites via GSK3β/Sgg negatively regulates tau binding to microtubules

**Fig 2:**
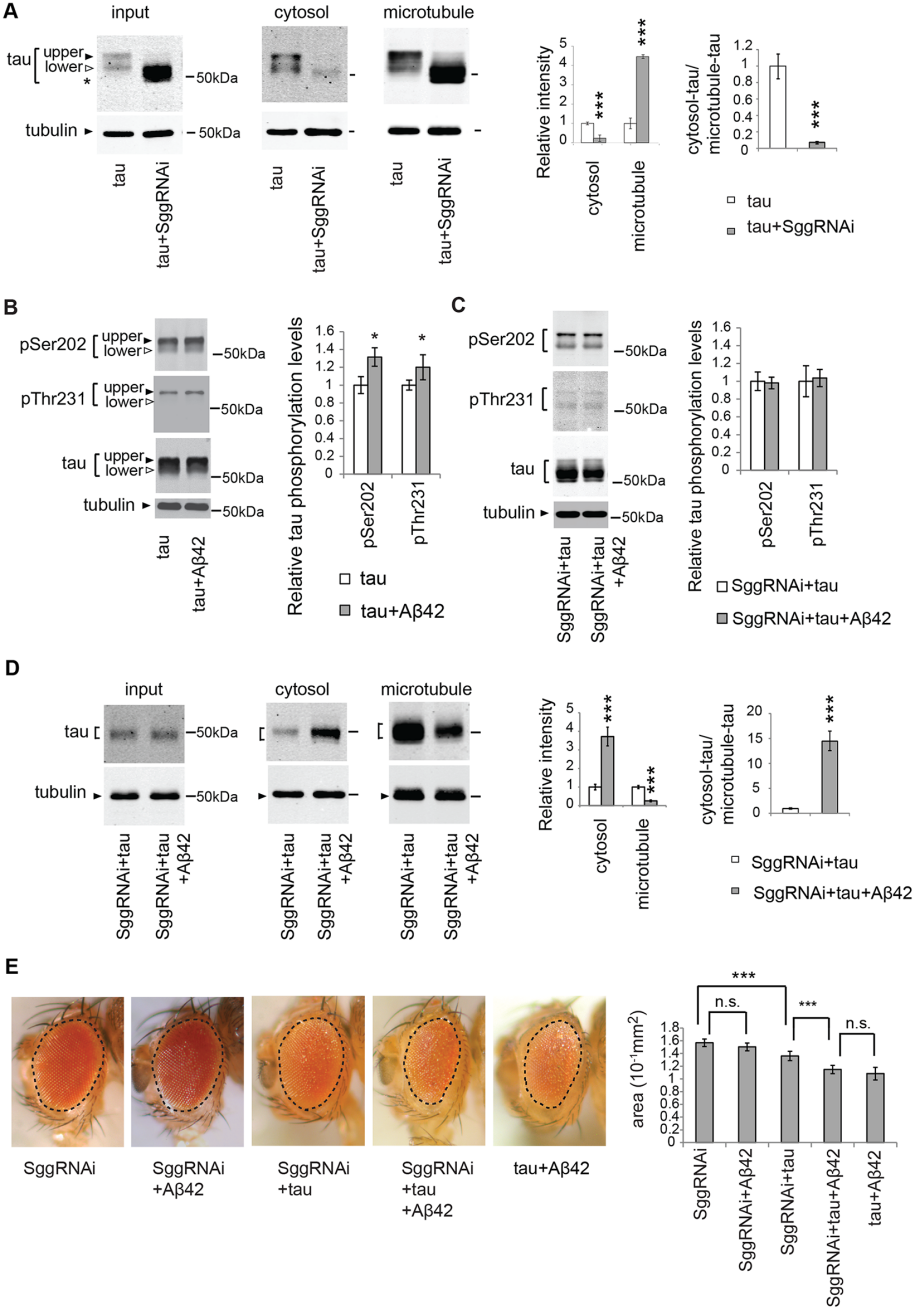
Knockdown of GSK3β/Sgg is not sufficient to suppress either mislocalization of tau to the cytosol or Aβ42-induced augmentation of tau toxicity. (A) GSK3β/Sgg negatively regulates tau binding to microtubules. RNAi-mediated knockdown of GSK3/Sgg shifts all tau species to lower apparent molecular weights, and the resultant species migrate faster than the original tau_lower_ species (indicated as asterisk). The levels of tau and tubulin in the lysate before sedimentation (input), in the supernatant (cytosol) and in the pellet containing microtubules (microtubule) were analyzed by western blotting by using anti-tau antibody. The same amount of proteins from each genotype was loaded. Mean ± SD, n = 4; **, *p* < 0.01, ***, *p* < 0.005 by Student's t-test. Representative blots are shown. (B) Aβ42 increased the levels of tau phosphorylated at Ser202 and those phosphorylated at Thr231. Western blots of fly heads expressing tau alone (tau) or that co-expressing tau and Aβ42 (tau+Aβ42) with anti-phospho-Ser202 antibody (pSer202), anti-phospho-Thr231 antibody (pThr231), and anti-tau antibody. Tubulin was used as a loading control. Mean ± SD, n = 5; *, *p* < 0.05 by Student's t-test. Representative blots are shown. (C) Expression of Aβ42 did not increase tau phosphorylation at either of Ser202 and Thr231 in the Sgg knockdown background. Western blots of fly heads expressing Sgg RNAi tau and (SggRNAi+tau) or that co-expressing Sgg RNAi, tau and Aβ42 (SggRNAi+tau+Aβ42) with anti-phospho-Ser202 antibody (pSer202), anti-phospho-Thr231 antibody (pThr231), and anti-tau antibody. Tubulin was used as a loading control. Mean ± SD, n = 5; no significant difference by Student's t-test (*p* > 0.05). Representative blots are shown. (D) Aβ42 causes an increase in tau levels in the cytosol fraction and reduction in tau levels in the microtubule fraction in the Sgg knockdown background. The levels of tau and tubulin in fly heads expressing Sgg RNAi and tau (SggRNAi+tau) or that co-expressing Sgg RNAi, tau and Aβ42 (SggRNAi+tau+Aβ42) before sedimentation (input), in the supernatant (cytosol) and in the pellet containing microtubules (microtubule) were analyzed by western blotting with anti-tau and anti-tubulin. Mean ± SD, n = 4; ***, *p* < 0.005 by Student's t-test. Representative blots are shown. (E) Aβ42 enhances tau-induced retinal degeneration in the Sgg knockdown background (compare SggRNAi+tau and SggRNAi+tau+Aβ42). Mean ± SE, N = 6–8, asterisks indicate significant differences in the surface area of the external eye (***, *p* < 0.005, n.s., not significant (*p* > 0.05) by one-way ANOVA with Tukey's post hoc test). Transgene expression was driven by gmr-GAL4.

Phosphorylation of tau negatively influences its ability to bind microtubules [25–27, 51, 52]. In *Drosophila*, tau phosphorylation at proline-directed Ser and Thr (SP/TP) sites decreases tau binding to microtubules, whereas replacing these phosphorylation sites with unphosphorylatable Ala significantly increases binding [25, 26]. In fly neurons, many of these SP/TP sites are phosphorylated by a fly homolog of GSK3 called Sgg [26, 53, 61]. In line with these reports, we found that RNAi-mediated knockdown of GSK3/Sgg significantly reduced tau phosphorylation at these SP/TP sites (S5 Fig); as a result, tau is detected as a single band with faster migration speed than tau_lower_ (Fig 2A, input, asterisk). *In vivo* microtubule-binding assays revealed that most tau was recovered in the microtubule fraction in the Sgg knockdown background (Fig 2A). These results indicate that, in this fly model, tau phosphorylation at SP/TP sites by GSK3β/Sgg plays an important role in tau binding to microtubules.

### Knockdown of GSK3β/Sgg is not sufficient to suppress either mislocalization of tau to the cytosol or Aβ42-induced augmentation of tau toxicity

As we reported previously, Aβ42 increased the levels of tau phosphorylated at Ser202 (pSer202) and Thr231 (pThr231) ([58] and Fig 2B). We found that, in the retina with RNAi-mediated GSK3β/Sgg knockdown, expression of Aβ42 did not increase the levels of tau phosphorylated at either of these residues (Fig 2C). These results suggest that increases in the levels of phosphorylated tau mediated by GSK3β/Sgg might contribute to Aβ42-induced detachment of tau from microtubules.

We tested whether blocking tau phosphorylation at SP/TP sites by GSK3/Sgg was sufficient to suppress Aβ42-mediated mislocalization of tau from the microtubule fraction to the cytosol fraction. *In vivo* microtubule-binding assays revealed that, in the GSK3/Sgg knockdown background, co-expression of Aβ42 was still capable of increasing the level of tau in the cytosolic fraction and decreasing the level in the microtubule fraction (Fig 2D). These results suggest that in the presence of Aβ42, even hypophosphorylated tau species that are normally bound to microtubules (Fig 2A) were mislocalized to the cytosolic fraction.

We next investigated whether RNAi-mediated knockdown of GSK3/Sgg suppressed Aβ42-induced augmentation of tau toxicity. Even in the GSK3/Sgg knockdown background, Aβ42 still promoted tau-mediated neurodegeneration, as indicated by smaller eye size in flies co-expressing tau and Aβ42 relative to those expressing tau alone (Fig 2E, compare SggRNAi+tau and SggRNAi+tau+Aβ42). In addition, GSK3/Sgg knockdown did not suppress neurodegeneration caused by tau and Aβ42 (Fig 2E, compare tau+Aβ42 and SggRNAi+tau+Aβ42). Under this experimental condition, expression of neither Aβ42 alone nor Aβ42 with GSK3β/Sgg RNAi caused a reduction in eye size (S6 Fig), suggesting that the observed augmentation of tau toxicity was not simply due to additive effects (Fig 2E).

Taken together, these results suggest that blocking tau phosphorylation at GSK3/Sgg sites is not sufficient to suppress either mislocalization of tau to the cytosol or Aβ42-induced augmentation of tau toxicity.

### Blocking tau phosphorylation at Ser262/356 with unphosphorylatable alanine substitutions preferentially reduces the levels of tau_lower_ and partially suppresses the Aβ42-mediated increase in tau mislocalization from the microtubule to the cytosol

**Fig 3:**
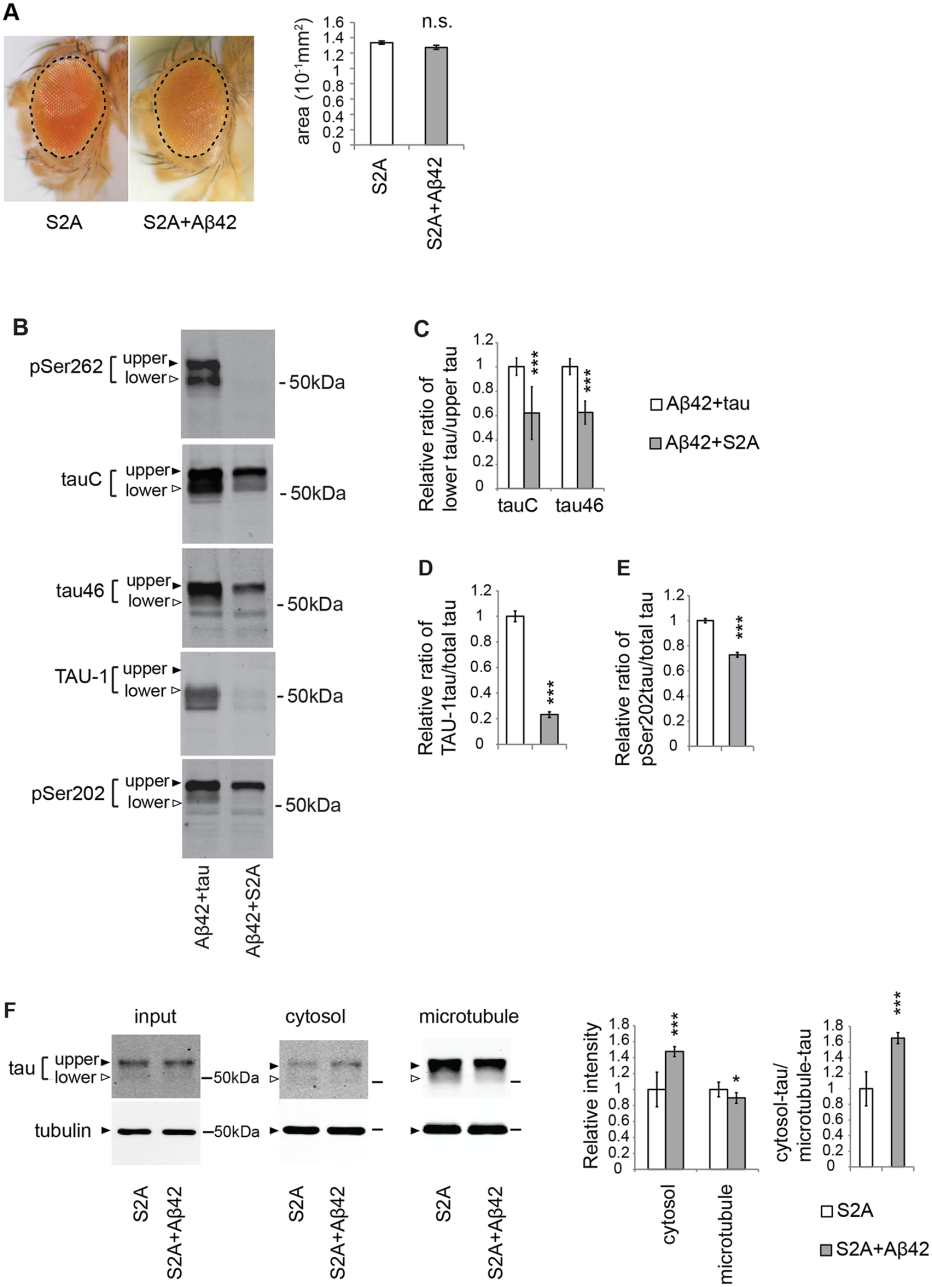
Blocking tau phosphorylation at Ser262/356 with unphosphorylatable alanine substitutions (S2A) preferentially reduces the levels of tau_lower_ and partially suppresses the Aβ42-mediated increase in tau mislocalization from the microtubule to the cytosol. (A) Expression of either S2Atau (S2A) alone or co-expression of S2Atau and Aβ42 (S2A+Aβ42) does not cause eye degeneration. No significant difference in the surface area of the external eyes between S2A and S2A+ Aβ42 (mean ± SE, n = 6–8, N.S., not significant (*p* > 0.05) by one-way ANOVA). Transgene expression was driven by gmr-GAL4. (B) S2Atau shows different phosphorylation profiles compared to wild-type tau. Wild-type tau or S2Atau were co-expressed with Aβ42 (tau+Aβ42 and Aβ42+S2A, respectively) and subjected to western blotting with pan-tau antibody (tauC and tau46) or antibodies that recognize phosphorylation status of tau at the specific sites (pSer262, TAU-1 and pSer202). (C) The ratio of signal intensities of tau_lower_ to tau^upper^ detected by tauC. (D) The ratio of signal intensities of TAU-1 blot to tauC blot. Mean ± SD, n = 5; ***, *p* < 0.005 by Student's t-test. (E) The ratio of signal intensities of pSer202 blot to tauC blot. Mean ± SD, n = 5; ***, *p* < 0.005 by Student's t-test. (F) Aβ42 increased the level of S2Atau in the cytosol and decreased those in the microtubule fraction, while the effects were smaller than those in wild-type tau. Mean ± SD, n = 4; *, *p* < 0.05, ***, *p* < 0.005 compared by Student's t-test. Representative blots are shown. Transgene expression was driven by gmr-GAL4.

Previous studies from several groups, including ours, showed that substitution of two Ser residues at 262 and 356 in tau to unphosphorylatable Ala (S2A) suppressed Aβ42-induced augmentation of tau toxicity ([58] and Fig 3A). These mutations abolished tau phosphorylation at Ser262 (Fig 3B, pSer262). Moreover, the SDS-PAGE migration pattern of S2A tau differed from that of wild-type tau.

As described above (Fig 1 and S3 Fig), wild-type tau proteins expressed in fly eyes are detected as two major bands reflecting different levels of phosphorylation (Fig 3B, Aβ42+tau in tauC). By contrast, S2A tau exhibited only a single major band corresponding to tau^upper^ (Fig 3B, Aβ42+S2A in tauC). Western blotting with the pan-tau antibodies tauC and tau46, which were raised against different epitopes and detect tau_lower_ to tau^upper^ with slightly different sensitivities, yielded similar results. To quantify this difference, we calculated the ratio of signal intensities of tau_lower_ to tau^upper^ and found that S2A tau had a lower ratio of tau_lower_ to tau^upper^ than wild-type tau (Fig 3C). In addition, western blotting with TAU-1 tau antibody, which specifically recognizes tau protein without phosphorylation at Ser194, Ser195, Ser198, and Ser202, and only detected tau_lower_ (Fig 1C), revealed that the level of TAU-1-positive tau was significantly reduced in S2A tau relative to wild-type tau (Fig 3B and 3D). These results suggest that less phosphorylated forms of tau (tau_lower_) were preferentially reduced in S2A tau.

**Fig 4:**
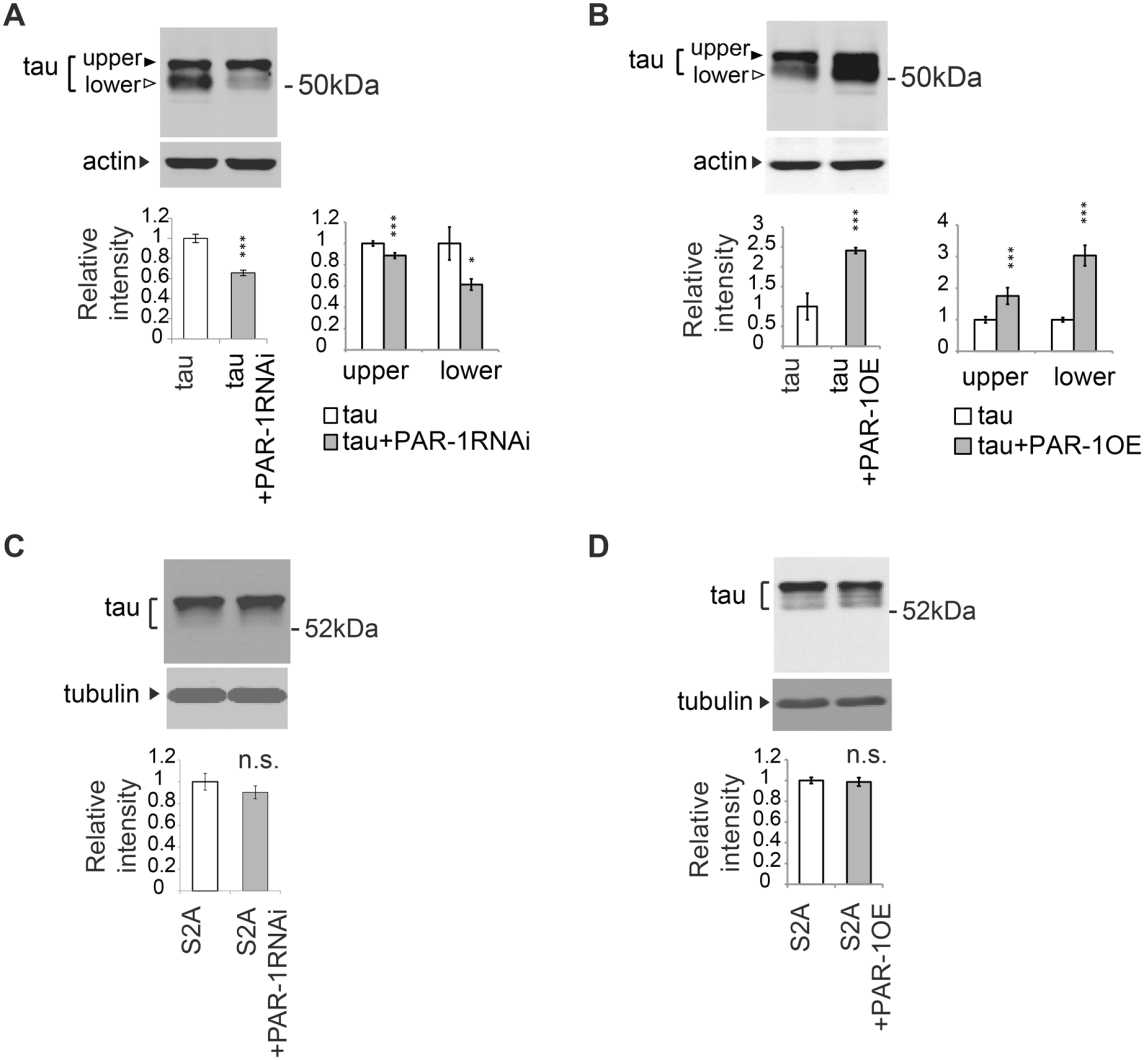
PAR-1 stabilizes less phosphorylated forms of tau (tau_lower_) through phosphorylation at Ser262 and Ser356. (A) PAR-1 knockdown reduces the levels of tau with more prominent effect on tau_lower_. Western blots of fly heads expressing tau (tau) or that co-expressing tau and PAR-1RNAi (tau+PAR-1RNAi) with anti-tau antibody. (B) PAR-1 overexpression increases the levels of tau with more prominent effect on tau_lower_. Western blots of fly heads expressing tau (tau) or that co-expressing tau and PAR-1 (tau+PAR-1OE) with anti-tau antibody. (C) PAR-1 knockdown does not affect S2Atau levels. Western blots of fly heads expressing S2Atau (S2A) or that co-expressing S2Atau and PAR-1RNAi (S2A+PAR-1RNAi) with anti-tau antibody. (D) PAR-1 overexpression does not affect S2Atau levels. PAR-1 knockdown does not affect S2Atau levels. Western blots of fly heads expressing S2Atau (S2A) or that co-expressing S2Atau and PAR-1 (S2A+PAR-1OE) with anti-tau antibody. Tubulin or actin was used as loading control. Mean ± SD, n = 4–5; *, *p* < 0.05, ***, *p* < 0.005, n.s., not significant (*p* > 0.05) by Student's t-test. Representative blots are shown. Transgene expression was driven by gmr-GAL4.

Because tau_lower_ represents less phosphorylated forms of tau, S2A mutation might reduce the relative ratio of tau_lower_ to tau^upper^ by promoting tau phosphorylation. However, this was not the case: when the phosphorylation levels of S2A tau at Ser202 were normalized against total levels of S2A tau, they were not elevated; instead, they were reduced relative to those of wild-type tau (Fig 3B and 3E). These results suggest that tau phosphorylation at Ser262 and Ser356 stabilizes less phosphorylated forms of tau (tau_lower_) but does not promote tau phosphorylation at SP/TP sites (see also Fig 4).

Next, we analyzed the effects of S2A mutation on Aβ42-mediated mislocalization of tau to the cytoplasm using an *in vivo* microtubule-binding assay. As described above, the levels of tau_lower_ were diminished in both the cytosolic and microtubule fractions. Moreover, although Aβ42 still increased the level of S2A tau^upper^ in the cytosol and decreased that in the microtubule fraction (Fig 3F), the effects were smaller than those in wild-type tau: Aβ42 caused a 3-fold increase in the level of wild-type tau^upper^ in the cytosol (Fig 1B), but only a 1.6-fold increase in the level of S2A tau^upper^ (Fig 3F, right).

Taken together, these data demonstrate that S2A mutation preferentially reduces the levels of tau_lower_, and partially suppresses the Aβ42-mediated increase in the levels of tau mislocalized from the microtubule to the cytosol.

### Tau phosphorylation at Ser262 and Ser356 via PAR-1 is critical for stabilization of tau_lower_

*Drosophila* PAR-1 kinase, a functional homolog of MARKs, is the major kinase of human tau at Ser262 and Ser356 expressed in fly eye and brain [53, 63], and RNAi-mediated knockdown of PAR-1 effectively decreases tau phosphorylation at Ser262 and Ser356 [63, 70]. To further elucidate the role of tau phosphorylation at Ser262 and Ser356 in the stabilization of tau, we asked whether knockdown of PAR-1 would also decrease the levels of less phosphorylated forms of tau (tau_lower_), as the S2A mutation did. Knockdown of PAR-1 significantly reduced the levels of tau_lower_ (Fig 4A), whereas overexpression of PAR-1 preferentially increased the levels of tau_lower_ (Fig 4B). The effects on the levels of tau^upper^ were less prominent. Importantly, PAR-1-mediated increases in tau levels required the presence of the Ser262 and Ser356 phosphorylation sites in tau. Knockdown of PAR-1 did not decrease the levels of S2A tau (Fig 4C), whereas co-expression of PAR-1 did not increase the levels of S2A tau (Fig 4D).

Expression of tau in the brain neurons has been reported to cause structural and functional abnormalities in *Drosophila* [53, 58, 71, 72]. Pan-neuronal expression of tau in the central nervous system affects development of the mushroom body structure in the brain [53, 58, 71–73], and tau expression in otherwise structurally intact adult neurons causes learning deficits [73]. Blocking tau phosphorylation at Ser262 and Ser356 suppresses these tau-induced defects [58, 71, 73], indicating that tau phosphorylation at Ser262 is critical for tau toxicity also in the brain neurons. To ask whether blocking tau phosphorylation at Ser262 and Ser356 also reduces tau levels in the brain neurons, we expressed tau by using the pan-neuronal driver elav-Gal4 and tested the effect of PAR-1 knockdown on tau levels. PAR-1 knockdown reduced the levels of tau, (S7 Fig), indicating that reduction in tau toxicity caused by blocking tau phosphorylation at Ser262 and Ser356 is concomitant with reductions in tau levels in the brain neurons as well.

Taken together, these results indicate that PAR-1 stabilizes less phosphorylated forms of tau (tau_lower_) through phosphorylation at Ser262 and Ser356.

### PAR-1/MARK mediates the increase in the level of tau phosphorylated at Ser262 caused by Aβ42, and knockdown of PAR-1/MARK markedly decreases the levels of tau_lower_

**Fig 5:**
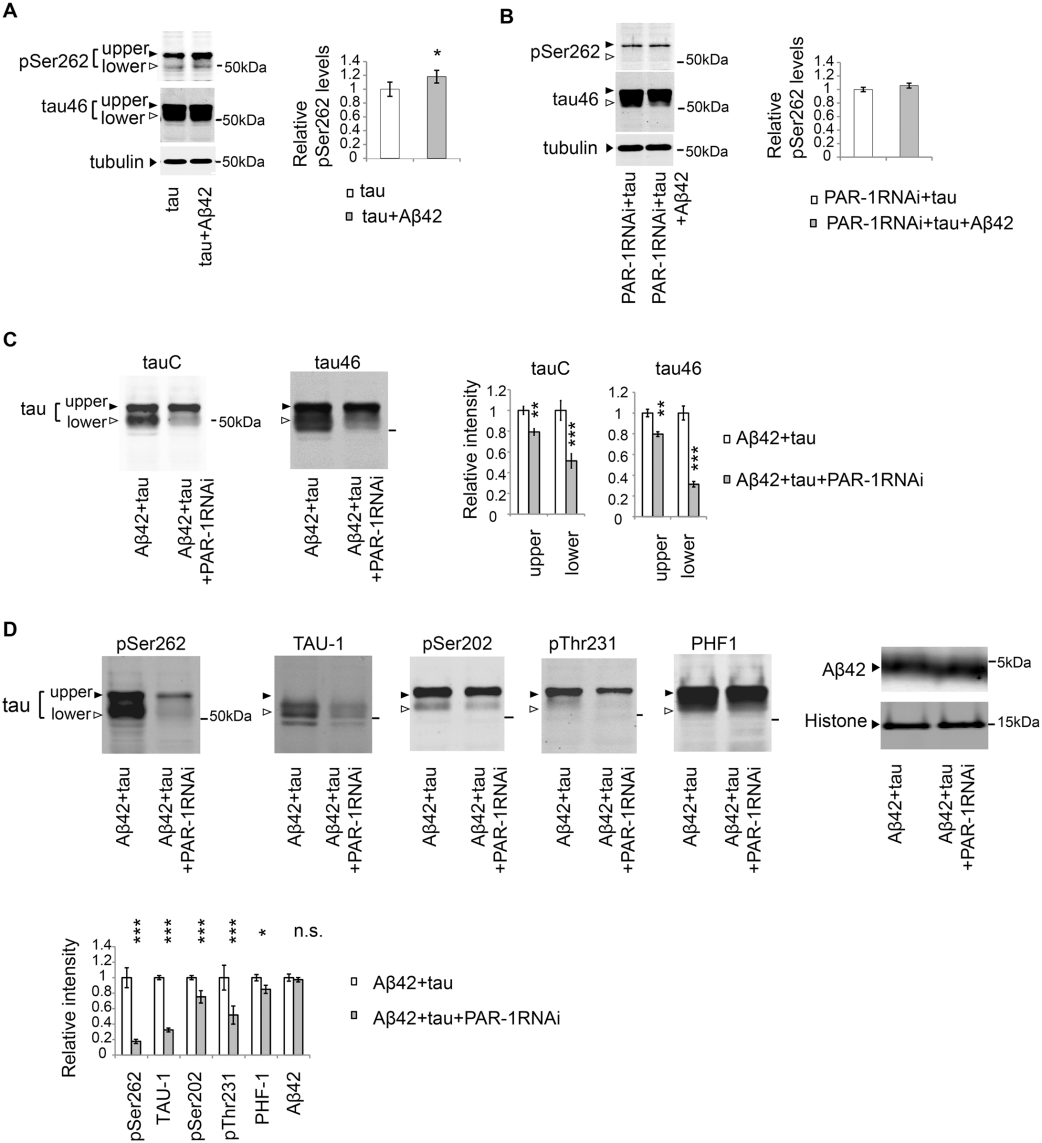
PAR-1 mediates the increase in the level of tau phosphorylated at Ser262 caused by Aβ42, and knockdown of Par-1/MARK markedly decreases the levels of tau_lower_. (A) Aβ42 increased the levels of tau phosphorylated at Ser262. Western blots of fly heads expressing tau alone (tau) or that co-expressing tau and Aβ42 (tau+Aβ42) with anti-phospho-Ser262 antibody (pSer262) or anti-tau (tau46). Tubulin was used as a loading control. Mean ± SD, n = 5; *, *p <* 0.05 by Student's t-test. Representative blots are shown. (B) Expression of Aβ42 did not increase tau phosphorylation at Ser262 in the PAR-1 knockdown background. Western blots of fly heads expressing PAR-1 RNAi and tau (PAR-1RNAi+tau) or that co-expressing PAR-1RNAi, tau and Aβ42 (PAR-1RNAi+tau+Aβ42) with anti-phospho-Ser262 antibody (pSer262) and anti-tau antibody (tau46). Tubulin was used as a loading control. Mean ± SD, n = 5; no significant difference by Student's t-test (*p* > 0.05). Representative blots are shown. (C-D) PAR-1 knockdown reduces the levels of tau with more prominent effect on the levels of tau_lower_ than those of tau^upper^. Western blots of fly heads expressing Aβ42 and tau (Aβ42+tau) or that co-expressing Aβ42, tau and PAR-1 RNAi (Aβ42+tau+PAR-1RNAi) with pan-tau antibody (tauC and tau46), antibodies that recognize phosphorylation status of tau at the specific sites (pSer262, TAU-1, pSer202, pThr231, PHF-1) or anti-Aβ antibody (Aβ42). Histone was used as loading control. Mean ± SD, n = 5; *, *p* < 0.05, **, *p* < 0.01, ***, *p*<0.005. Representative blots are shown. Transgene expression was driven by gmr-GAL4.

The level of tau phosphorylated at Ser262 is elevated in the Aβ42 fly retina [58] (Fig 5A), and this increase was suppressed in the PAR-1 knockdown background (Fig 5B). These results suggest that tau is stabilized via phosphorylation at Ser262 by PAR-1 in the Aβ42 fly retina.

We next tested whether knockdown of PAR-1 would reduce the levels of less phosphorylated forms of tau (tau_lower_) in the presence of Aβ42. Western blot analyses using the pan-tau antibodies tauC and Tau46 revealed that PAR-1 knockdown reduced the levels of tau (Fig 5C, tauC/Tau46), and the reductions in the levels of tau_lower_ were more prominent than those of tau^upper^ (Fig 5C, tauC/Tau46,). The levels of co-expressed Aβ42 were not influenced by PAR-1 knockdown (Fig 5D), suggesting that PAR-1 knockdown did not simply increase protein degradation in a non-specific manner.

To further characterize the reduction of tau phosphorylation in the PAR-1 knockdown background, we used a panel of antibodies (Fig 1C) that could distinguish the phosphorylation status of tau at AD-associated sites. Western blot analyses using these antibodies confirmed that PAR-1 knockdown significantly decreased the levels of tau phosphorylated at Ser262. PAR-1 knockdown also caused a prominent reduction in the levels of TAU-1-positive, tau_lower_ (Fig 5D). PAR-1 knockdown also reduced the levels of pThr231-positive tau, whereas the PAR-1 knockdown-mediated reductions in the levels of pSer202- and PHF-1-positive tau were less prominent (Fig 5D).

A previous study reported that blocking tau phosphorylation at Ser262/356 decreased the levels of tau phosphorylation at SP/TP sites including Ser202 [53]. In that study, it has been suggested that tau phosphorylation at Ser262/356 primes the protein for subsequent phosphorylation events. In this model, blocking tau phosphorylation at Ser262/356 sites should abolish the downstream phosphorylation events and a larger proportion of tau protein should be non-phosphorylated at SP/TP sites, including Ser202. However, western blot analysis using the TAU-1 antibody, which specifically recognizes tau lacking phosphorylation at Ser202, revealed that blocking tau phosphorylation at Ser262/356 by either by PAR-1 knockdown (Fig 5D) or introduction of the S2A mutation (Fig 3B), did not increase, but rather significantly decreased, the levels of tau species that are not phosphorylated at Ser202. These data suggest that tau phosphorylation at Ser262/356 rather regulates the stability of tau_lower_ species.

Taken together, these results suggest that blocking tau phosphorylation at Ser262/356 by PAR-1 knockdown preferentially reduces the levels of tau_lower_ also in the presence of Aβ42.

### Blocking tau phosphorylation at SP/TP sites by knockdown of GSK3/Sgg does not reduce the levels of tau

Hyperphosphorylation of tau at SP/TP sites is thought to promote aggregation and accumulation of tau [61, 74, 75]. A previous report showed that tau phosphorylation at Ser262 and Ser356 by PAR-1 plays a priming role in subsequent tau phosphorylation at SP/TP sites, and that knockdown of PAR-1 reduces the level of tau phosphorylated at these sites [53] (also see Fig 5D). Moreover, a mammalian homolog of PAR-1, MARK, positively regulates the activity of GSK3β and may promote tau phosphorylation at SP/TP sites [26, 53, 61]. Consistent with this, phosphorylation levels of GSK3/Sgg at Ser9, which negatively regulates kinase activity [76], were increased by PAR-1 knockdown (S8 Fig), suggesting that PAR-1 may positively regulate GSK3/Sgg activity in the fly retina as well. Because tau phosphorylation at SP/TP sites is resistant to proteolytic degradation [77], these observations raise the possibility that knockdown of PAR-1 might reduce tau phosphorylation at SP/TP sites, thereby promoting the degradation of less phosphorylated forms of tau.

**Fig 6:**
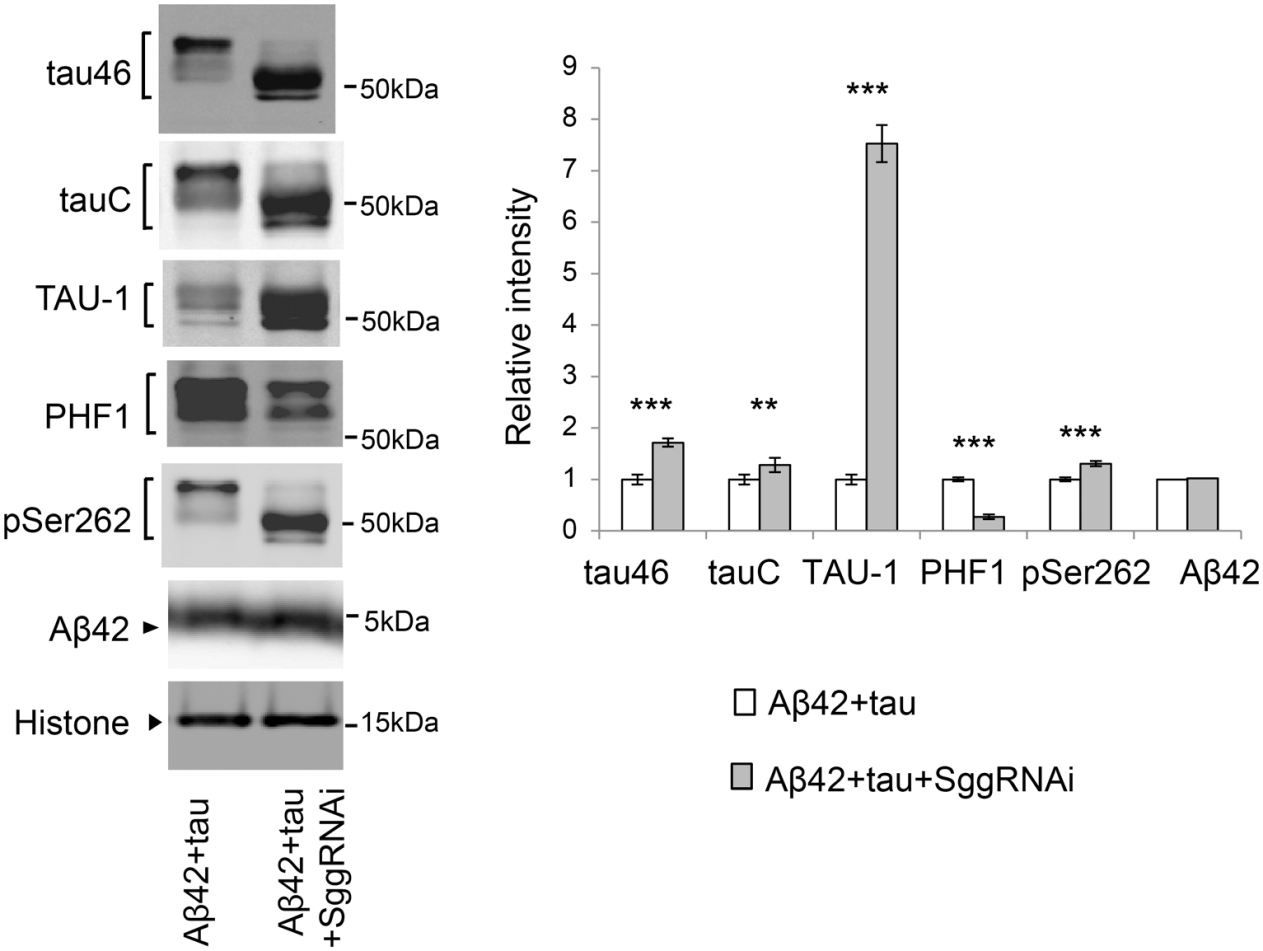
Blocking tau phosphorylation at SP/TP sites by Sgg knockdown does not reduce the levels of tau. Western blots of fly heads expressing Aβ42 and tau (Aβ42+tau) or that co-expressing Aβ42, tau and Sgg RNAi (Aβ42+tau+SggRNAi) with pan-tau antibody (tauC and tau46), antibodies that recognize phosphorylation status of tau at the specific sites (TAU-1, PHF-1 and pSer262) or anti-Aβ antibody (Aβ42). Histone was used as loading control. Mean ± SD, n = 5; **, *p* < 0.01, ***, *p* < 0.005. Representative blots are shown. Transgene expression was driven by gmr-GAL4.

To investigate this possibility, we next asked whether blocking tau phosphorylation at SP/TP sites by knockdown of GSK3/Sgg was sufficient to reduce tau levels. Western blot analyses using a panel of anti-pan-tau, anti-non-phospho-tau, and anti-phospho-tau antibodies confirmed that knockdown of GSK3β/Sgg significantly decreased tau phosphorylation levels at SP/TP sites (Fig 6). Migration speed of tau (Fig 6, tau46/tauC) and the level of non-phosphorylated tau were elevated (Fig 6, TAU-1), whereas the level of PHF1-positive tau was reduced (Fig 6, PHF1). These results suggest that knockdown of GSK3β/Sgg significantly decreased tau phosphorylation levels at SP/TP sites. Under this condition, the levels of tau detected with pan-tau antibodies tau46 and tauC, TAU-1-positive tau, and tau phosphorylated at Ser262 were not reduced, but were instead slightly elevated (Fig 6, tau46, tauC, TAU-1, and pSer262). These results suggest that tau phosphorylation at SP/TP sites by GSK3/Sgg does not play a major role in stabilization of tau in the fly retina.

### Stabilization of tau through phosphorylation at Ser262 and Ser356 contributes to the Aβ42-induced increase in the level of tau phosphorylated at Thr231

**Fig 7:**
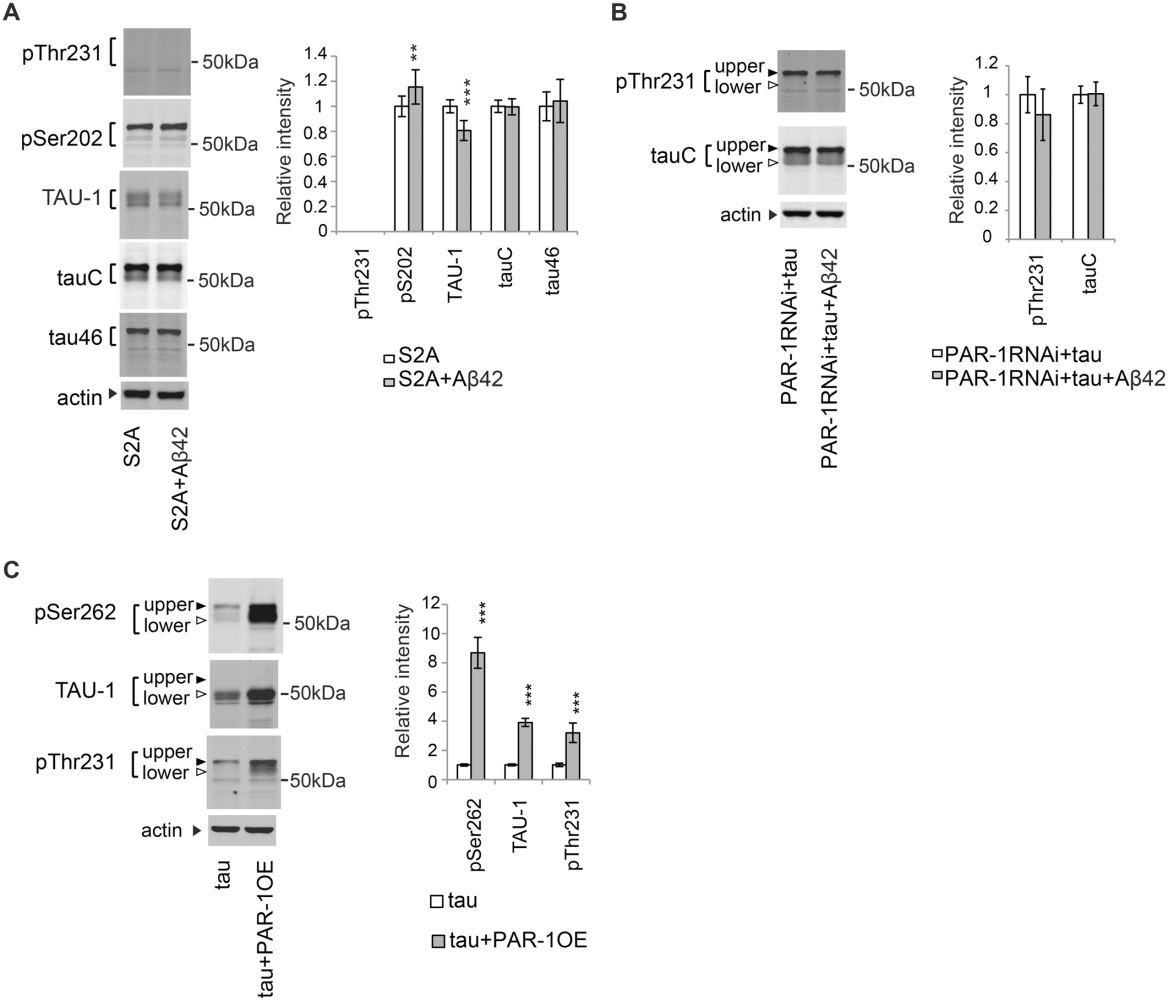
Stabilization of tau through phosphorylation at Ser262 and Ser356 contributes to the Aβ42-induced increase in the level of tau phosphorylated at Thr231. (A) Aβ42 does not increase the levels of S2Atau phosphorylated at Thr231. Western blots of fly heads expressing S2Atau (S2A) or that co-expressing S2Atau and Aβ42 (S2A+Aβ42) with antibodies that recognize phosphorylation status of tau at the specific sites (pThr231, pSer202, and TAU-1) or pan-tau antibodies (tauC and tau46). (B) Aβ42 does not increase the levels of tau phosphorylated at Thr231 in the PAR-1 knockdown background. Western blots of fly heads expressing PAR-1RNAi and tau (PAR-1RNAi+tau) or that co-expressing PAR-1RNAi, tau and Aβ42 (PAR-1RNAi+tau+Aβ42) with anti-phospho-Thr231 antibody (pThr231) or pan-tau antibody (tauC). (C) PAR-1 overexpression increases the levels of tau phosphorylated at Thr231. Western blots of fly heads expressing tau (tau) or that co-expressing PAR-1 and tau (tau+PAR-1OE) with anti-phospho-Ser262 antibody (pSer262), TAU-1 or anti-phospho-Thr231 antibody (pThr231). Actin was used as loading control. Mean ± SD, n = 5; **, *p <* 0.01, ***, *p* < 0.005. Representative blots are shown. Transgene expression was driven by gmr-GAL4.

Because tau phosphorylation at Ser262 was increased by Aβ42 [58] (Fig 5A), we asked whether stabilization of tau through tau phosphorylation at Ser262 and Ser356 contributed to the Aβ42-mediated increase in the level of tau phosphorylated at AD-related SP/TP sites, Ser202 and Thr231 [58] (Fig 2B). Introduction of unphosphorylatable Ala at the Ser262 and Ser356 sites in tau (S2A) blocked Aβ42-mediated increases in the levels of tau phosphorylated at Thr231 (Fig 7A). Similar effects were also observed upon knockdown of PAR-1 (Fig 7B). By contrast, Aβ42 increased the level of S2A tau phosphorylation at Ser202 (Fig 7A: pS202 level was elevated, whereas TAU-1 level was reduced).

To further validate the dependence of tau phosphorylation at Thr231 on Ser262/356 phosphorylation, we investigated whether upregulation of tau phosphorylation at Ser262/356 by PAR-1 was sufficient to increase the levels of tau phosphorylated at Thr231. Overexpression of PAR-1 significantly increased the levels of tau phosphorylation levels at Ser262 as expected (Fig 7C, pSer262). In addition, the levels of TAU-1-positive tau were markedly increased (Fig 7C, TAU-1), consistent with its stabilizing effect on tau_lower_ (Fig 4B). Under these conditions, the level of tau phosphorylated at Thr231 was significantly increased (Fig 7C, pThr231).

Because GSK3/Sgg is required for the Aβ42-mediated increase in the levels of tau phosphorylated at Ser202 and Thr231 (Fig 2C), we also investigated whether Aβ42 increased GSK3/Sgg activity in this model. Phosphorylation levels of GSK3/Sgg at Ser9 sites, which negatively correlate with GSK3/Sgg activity, were not significantly altered by Aβ42 (S9 Fig), suggesting that Aβ42-mediated increases in the levels of tau phosphorylated at Ser202 and Thr231 were not due to global activation of GSK3/Sgg. Indeed, the levels of tau phosphorylated at other GSK3/Sgg-target sites were not altered by Aβ42 in this model [58].

**Fig 8:**
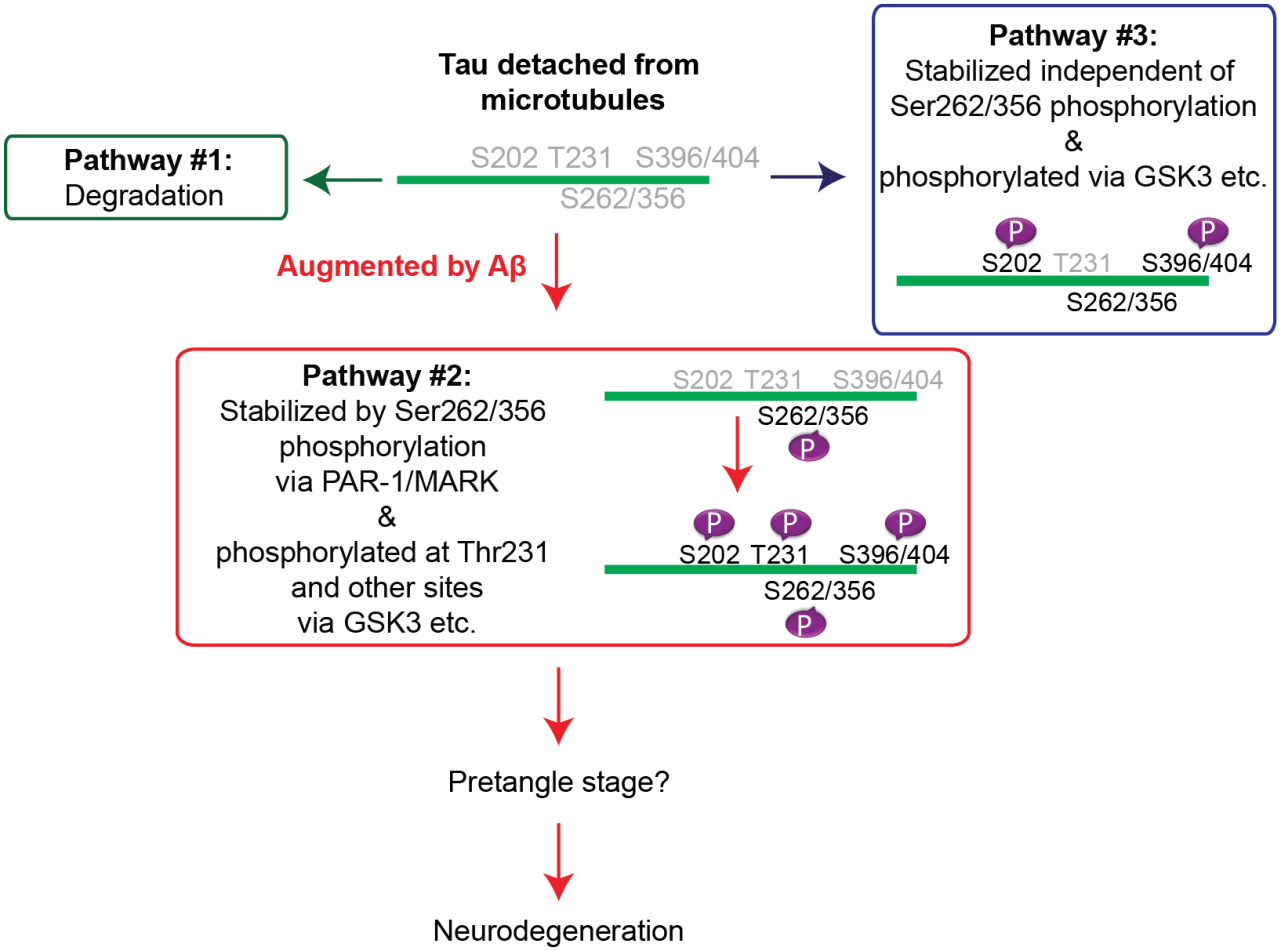
Working model of the early phase of tau mismetabolism.

Taken together, these results suggest that tau phosphorylation at Thr231 depends strongly on tau phosphorylation at Ser262 and Ser356 by PAR-1/MARK, and stabilization of tau may contribute to the increase in the levels of tau phosphorylated at an AD-related SP/TP site, Thr231, caused by Aβ42. These data also suggest that increases in the levels of tau phosphorylated at other AD-related SP/TP sites, such as Ser202, might be mediated by mechanisms distinct from tau species phosphorylated at Thr231 (model in Fig 8).

## Discussion

### Tau phosphorylation at Ser262/356 via PAR-1/MARK stabilizes microtubule-unbound tau at an initial step of tau mismetabolism

Tau phosphorylation at Ser262/356 is detected in the pre-tangle stage of neurons in AD brains [78]. Several reports suggest that tau phosphorylation at these sites initiates mismetabolism of tau by increasing the levels of microtubule-unbound free tau [66]. Ser262 and Ser356 are located in the microtubule-binding domain, and phosphorylation at these sites decreases tau binding to microtubules [18, 19, 25, 26, 50, 51]. In addition, tau phosphorylation at Ser262/356 is reported to promote tau phosphorylation at other SP/TP sites [53, 55], which may also decrease tau binding to microtubules and increases the levels of microtubule-unbound tau. This study demonstrates a novel mechanism by which tau phosphorylation at Ser262/356 via PAR-1/MARK regulates the levels of microtubule-unbound tau in the cytosol: tau phosphorylation at Ser262/356 sites preferentially stabilizes less phosphorylated forms of tau (tau_lower_). Importantly, blocking this stabilization of tau suppressed tau-mediated neurodegeneration and reduced the level of tau phosphorylated at the AD-associated residue Thr231 (Fig 7). Furthermore, these processes are involved in Aβ42-induced augmentation of tau toxicity (Fig 3), suggesting the relevance of this step to AD pathogenesis. Collectively, our data reveal a novel role for tau phosphorylation by PAR1/MARK at Ser262 and Ser356 in the stabilization of tau at an early step of mismetabolism, ultimately leading to tau toxicity.

### Microtubule-detached, tau_lower_ species contributes to the augmentation of tau toxicity caused by Aβ42

We demonstrate that blocking tau phosphorylation at AD-related Ser262/356 sites by introduction of the S2A mutation preferentially decreases the levels of tau_lower_ species and suppresses Aβ-mediated augmentation of tau toxicity (Fig 3). These results suggest that tau_lower_ species contribute to neurotoxicity in this experimental model. By contrast, blocking tau phosphorylation at SP/TP sites by RNAi-mediated knockdown of GSK3β/Sgg did not suppress Aβ42-mediated enhancement of tau toxicity (Fig 2). This observation suggests that tau phosphorylation by GSK3/Sgg is not required for promotion of tau toxicity by Aβ in this model. In addition, knockdown of Sgg significantly decreased phosphorylation levels of tau at SP/TP sites and shifted all tau species to lower–molecular weight regions (Fig 2); therefore, Aβ is capable of promoting tau toxicity under conditions in which the levels of tau^upper^ species are diminished.

It has been shown that both introduction of S2A mutation and Sgg knockdown reduce tau toxicity [26, 53], suggesting that multiple tau species mediate neurotoxicity in a context-dependent manner. From the results of this study, we cannot draw an explicit conclusion regarding which tau species, tau^upper^ or tau_lower_, is more toxic. In our model system, however, microtubule-detached tau_lower_ species contributed to the augmentation of tau toxicity caused by Aβ42, and tau phosphorylation at Ser262/356 played a critical role in the stabilization of tau_lower_ in this process.

Our results also suggest that the difference between tau_lower_ and tau^upper^ species is not simply due to different levels of phosphorylation at SP/TP sites mediated by GSK/Sgg. If it were the case, Sgg RNAi would increase the abundance of tau species qualitatively similar to the original tau_lower_ species and greatly promote neurodegeneration. However, we found that Sgg RNAi did not enhance the neurodegeneration phenotype (Fig 2E), suggesting that the original tau_lower_ species may be qualitatively different from tau species that are less phosphorylated at SP/TP sites as a consequence of Sgg knockdown. These results raise the possibility that the difference between the original tau_lower_ and tau^upper^ species includes not only phosphorylation status at GSK3-target sites but also additional post-transcriptional modifications and/or differences in cellular localization.

### How tau phosphorylation at Ser262/356 contributes to neurodegeneration

Our results show that blocking tau phosphorylation at Ser262/356 via PAR-1/MARK reduces tau levels and suppresses augmentation of tau-mediated neurodegeneration caused by Aβ42 (Fig 3), suggesting that elevated levels of tau phosphorylated at Ser262/356 promote neurodegeneration. Although the mechanisms underlying tau-mediated neurodegeneration remain unclear, one potential mechanism might involve disruption of protein degradation pathways. The majority of tau species (including tau lacking phosphorylation at Ser262/356) are degraded by the proteasome [20], while aggregated tau or tau phosphorylated at Ser262/356 is degraded by the autophagy-lysosome system [79–84]. This suggests that abnormal increases in the level of tau phosphorylated at Ser262/356 may interfere with or overload the autophagy-lysosome pathway. Consistent with this, the autophagic system and endosome/lysosome pathways are compromised in AD brains, and chronic disturbance of this system is thought to contribute to protein accumulation and neurodegeneration [85–87]. Alternatively, sustained activation of the autophagy pathway might shift the balance of proteostasis and disturb normal cellular functions, potentially causing neuronal dysfunction and degeneration. Supporting this hypothesis, the lysosomal system is upregulated in vulnerable cell populations in AD brains [88]. Further studies regarding the fate of tau phosphorylated at Ser262/356 may reveal the mechanisms underlying the compromised autophagy-lysosome system caused by tau and tau-mediated neurodegeneration in AD and other tauopathies.

### Blocking tau phosphorylation at Ser262/356 also reduces tau phosphorylated at Thr231

We found that the Aβ-induced increase in tau phosphorylation at Thr231 depends on phosphorylation at Ser262/356 (Fig 7). Interestingly, similar to tau phosphorylated at Ser262/356, tau phosphorylated at Thr231 is detected in the pre-tangle stage in AD brains [89], and is often found detached from microtubules *in vitro* and in cultured cells [51, 90]. These observations suggest that toxic tau species generated in the early stage of mismetabolism are phosphorylated at both Ser262/356 and Thr231. There are ample data showing the reduction and modification in tau phosphorylated at Thr231, which have been linked to suppression of tau toxicity [91–94]. The HDAC inhibitor nicotinamide specifically augments degradation of tau phosphorylated at Thr231 and suppresses tau-induced neurodegeneration in a transgenic mouse model [91]. Tau phosphorylated at Thr231 is isomerized by the cis-trans isomerase Pin-1, which protects against neurodegeneration [92–94]. Moreover, Thr231 and Ser262 may work in concert to promote the abnormal metabolism of tau and augment its toxicity. Phosphorylation of tau at both Thr231 and Ser262 achieves maximal inhibition of microtubule binding *in vitro* [95], and introduction of pseudophosphorylation at Thr231 and Thr212 with Ser262 augments tau toxicity in cultured cells [96] and transgenic *Drosophila* [97]. Our data suggest that targeting tau phosphorylated at Ser262/356 could reduce the levels of tau phosphorylated at Thr231, thereby suppressing tau toxicity associated with Thr231 phosphorylation. Further studies using mammalian model systems of tau toxicity are warranted to test this hypothesis.

In addition to Thr231, a recent report showed that tau phosphorylation at Ser238 was important for tau toxicity, and occupation of Ser262 precedes and was required for Ser238 phosphorylation [55, 71]. We investigated the possibility that tau phosphorylation at Ser238 might contribute to tau toxicity in this model. Consistent with the previous report, phospho-Ser238 tau was detected by western blot in the fly heads expressing tau in neurons under the control of the pan-neuronal elav promoter. However, fly heads expressing tau alone or co-expressing tau and Aβ in the retina under the control of the gmr-GAL4 driver did not exhibit a detectable phospho-Ser238 signal by western blot analyses (S10 Fig). This result suggests that, when tau is expressed in the fly retina, the levels of tau phosphorylated at Ser238 are very low.

### Tau phosphorylated at Ser262/356 is a potential novel therapeutic target

Current tau-lowering strategies include augmentation of protein degradation systems such as autophagy and the proteasome [87] and immunization against tau phosphorylated at disease-associated sites [98–106]. Pharmacological modulation of protein kinases that mediate hyperphosphorylation of tau, such as GSK3, Cdk5, and ERK2, also represents a feasible therapeutic strategy [107]. Inhibition of tau aggregation is another approach, and a number of small molecules that inhibit tau aggregation have been identified [108]. However, recent studies suggest that soluble, intermediate misfolded forms of tau, which may not be targeted by aggregation inhibitors, also exert neurotoxicity [109–111]. Thus, to effectively prevent the cascade of events leading to tau-mediated neurodegeneration, it is important to target toxic tau species at the earliest steps of abnormal metabolism. Our results suggest that tau phosphorylated at Ser262/356 represents a target for an effective strategy to reduce tau species in the early stage of mismetabolism, including soluble intermediate forms. Inhibition of MARK has been suggested as a possible strategy to specifically target tau phosphorylated at Ser262/356 [112].

### Several pathways in tau mismetabolism have been dissected

This study highlights the critical roles of tau phosphorylation at Ser262/356 in stabilization of tau and its contribution to tau-mediated neurodegeneration. Our data also suggest the existence of tau species whose stabilization is independent of tau phosphorylation at Ser262/356. Tau toxicity is likely to be qualitatively and quantitatively heterogeneous, possibly due to the diversity of tau species associated with distinct modifications, binding partners, and/or cellular locations [113]. Thus, targeting multiple pools of abnormal tau stabilized by distinct pathways could additively suppress tau-mediated toxicity. Further elucidation of the mechanisms underlying stabilization of tau *in vivo* may reveal additional therapeutic targets that could effectively lower tau levels, thereby counteracting complex tau toxicity in AD and other tauopathies.

## Materials and Methods

### Fly stocks

Flies were maintained in standard cornmeal media at 25°C. The transgenic fly lines carrying the human 0N4R tau, which has four tubulin-binding domains (R) at the C-terminal region and no N-terminal insert (N), include a kind gift from Dr. M. B. Feany (Harvard Medical School) [59] and the lines established following the standard method [63, 114] by using human 0N4R tau (a kind gift from Dr Mike Hutton (Mayo Clinic Jacksonville)). The UAS-Aβ42 and UAS-luciferase RNAi transgenic flies were previously described [63, 64, 114]. The transgenic fly line carrying UAS-S2Atau was established following the standard method [63, 114] by using human 0N4R tau (a kind gift from Dr Mike Hutton (Mayo Clinic Jacksonville)) with alanine mutation at Ser262 and Ser356 introduced by using the QuikChange site-directed mutagenesis kit (Stratagene, La Jolla, CA, USA). The elav-GAL4, GMR-GAL4, UAS-CD8GFP were obtained from the Bloomington Stock Center. UAS-PAR-1 RNAi is a gift from Dr. J. McDonard (Cleveland Clinic, Cleveland, USA) [70]. UAS-PAR-1 is a gift from Dr. Bingwei Lu (Stanford University) [115]. UAS-Sgg RNAi, UAS-mCherry RNAi (TRiP at Harvard Medical School) were obtained from Bloomington stock center. All experiments were performed using age-matched male flies, and genotypes are described in S1 Table.

### Western blotting

Western blotting was carried out as described previously [63]. Briefly, twenty fly heads for each genotype were homogenized in SDS-Tris-Glycine sample buffer, and the same amount of the lysate was loaded to each lane of multiple 10% Tris-Glycine gels and transferred to nitrocellulose membrane. The membranes were blocked with 5% milk (Nestle), blotted with the antibodies described below, incubated with appropriate secondary antibody and developed using ECL plus Western Blotting Detection Reagents (GE Healthcare) or imaging with an Odyssey system. One of the membranes was probed with anti-tubulin, and used as the loading control for other blots in each experiment. Anti-tau monoclonal antibody (Tau46, Zymed), anti-tau phospho-Ser262 (Biosource and AbCam), phospho-Thr231 (AT180, Thermo and Endogen), TAU1 (millipore), anti-GSK3 (Cell Signaling), anti-GSK3 phospho-Ser9/21 (Cell Signaling), anti-Aβ (6E10) (Signet, Covance), anti-tubulin (Sigma), anti-GFP (Clontech), anti-acetyl tubulin (Sigma), anti-tyrosinated tubulin (Sigma) were purchased. Anti-tau pS202 (CP13) and phospho-Ser396/404 (PHF1) was a kind gift from Dr. Peter Davis (Albert Einstein College of Medicine, USA), and anti-tau polyclonal antibody (tauC) was a kind gift from Dr. A. Takashima (National Center for Geriatrics and Gerontology, Japan) [116]. The signal intensity was quantified using ImageJ (NIH) or an Odyssey system. Western blots were repeated a minimum of three times with different animals and representative blots are shown. Flies used for Western blotting were 3–5 day-old after eclosion.

### Phosphatase treatment

Twenty fly heads for each genotype were homogenized in NEBuffer1 (50 mM HEPES, 100 mM NaCl, 2 mM DTT, 0.01% Brij 35, pH 7.5) supplemented with 1 mM MnCl_2_ (NEB) and proteinase inhibitor cocktail (Roche), and incubated with λ protein phosphatase (NEB) for 3h at 30°C, then subjected to western blotting as described above.

### *In vivo* microtubule-binding assay

Microtubule binding assay was performed using a previously reported [63]. Fifty heads from adult flies expressing the human tau protein with the gmr-GAL4 driver were collected and homogenized in 150 μl of Buffer-C+ [50 mM 4-(2-hydroxyethyl)-1-piperazineethanesulfonicacid pH 7.1, 1 mM MgCl2, 1 mM ethylene glycol tetraacetic acid, protease inhibitor cocktail (Roche), and phosphatase inhibitor cocktail (Sigma-Aldrich)] in the presence of taxol 20 μM (Sigma-Aldrich) diluted in dimethylsulfoxide. After centrifugation at 1,000× g for 10 min, aliquot of supernatant was subjected to western blotting as the “input fraction”. The remaining supernatant was layered onto a 2 volume cushion of buffer-C+ with 50% sucrose. After centrifugation at 100, 000× g for 30 min, 1/3 volume of the supernatant containing soluble tubulin was collected from the top of the tube as the cytosol fraction, and the pellet containing microtubule polymers and proteins bound to microtubules was resuspended in 150 μl of SDS-Tris-Glycine sample buffer. Protein concentration in each fraction was measured using the BCA Protein Assay Kit (Pierce). The same amount of protein was loaded to each lane of Tris-Glycine gels and analyzed by western blotting using anti-tau antibody (Tau46, Zymed) or anti-tubulin (Sigma). For quantification, the signal intensity in each lane was quantified with an Odyssey system.

### Histological analysis

Preparation of paraffin sections, hematoxylin and eosin staining, and analysis of neurodegeneration were described previously [63]. To analyze internal eye structure, heads of female flies were fixed in Bouin's fixative (EMS) for 48 hr at room temperature, incubated 24 hr in 50 mM Tris/150 mM NaCl, and embedded in paraffin. Serial sections (6 μm thickness) through the entire heads were prepared, stained with hematoxylin and eosin (Vector), and examined by bright-field microscopy. Images of the sections that include the lamina were captured with Insight 2 CCD Camera (SPOT), and vacuole area was measured using Image J (NIH). Heads from more than five flies (more than 10 hemispheres) were analyzed for each genotype.

### Statistics

Statistics was done with the JMP software (SAS) or R (R Development Core Team (2008)) with Student's t, or one-way ANOVA followed by Tukey-Kramer HSD.

## Supporting Information

S1 Fig Co-expression of Aβ42 promotes tau-mediated retinal degeneration. External eyes of flies expressing the gmr-gal4 driver alone (control), human tau (tau), tau and Aβ42 (tau+Aβ42) or Aβ42 (Aβ42). The surface areas of the external eyes are shown as mean ± SE (n = 6–8, one-way ANOVA, n = 5; ***, *p*<0.005, n.s., not significant (*p>*0.05)). Genotypes are as follows: (control) gmr-GAL4/+, (tau) gmr-GAL4/+;UAS-tau/+, (tau+Aβ42) gmr-GAL4/UAS-Aβ42;UAS-tau/+ and (Aβ42) gmr-GAL4/UAS-Aβ42. Transgene expression was driven by gmr-GAL4.
(TIF)

S2 Fig Co-expression of non-toxic proteins in the secretory pathway does not affect the levels of tau free from microtubules and those bound to microtubules. The levels of tau and tubulin in the lysate of fly heads expressing tau alone (tau) or co-expressing tau and non-toxic proteins in the secretory pathway CD8GFP (tau+CD8GFP) before sedimentation (input), in the supernatant (cytosol) and in the pellet containing microtubules (microtubule) were analyzed by western blotting by using anti-tau antibody. The same amount of proteins from each genotype was loaded. Expression of CD8GFP was confirmed by western blotting with anti-GFP antibody (CD8GFP). Mean ± SD, n = 5; *p* > 0.05 by Student's t-test. Representative blots are shown. Transgene expression was driven by gmr-GAL4. Genotypes are as follows: (tau) gmr-GAL4/+;UAS-tau/+ and (tau+CD8GFP) gmr-GAL4/UAS-CD8GFP;UAS-tau/+.
(TIF)

S3 Fig The differences between two major tau bands are related to their phosphorylation levels. Western blots of lysate of fly heads expressing tau with or without phosphatase treatment with anti-tau antibody. Following phosphatase treatment, the two tau bands (indicated by arrowheads) merged and were detected as a single faster-migrating band (indicated by asterisk). Transgene expression was driven by gmr-GAL4. The fly genotype is gmr-GAL4/+;UAS-tau/+.
(TIF)

S4 Fig Aβ42 expression does not affect the levels of acetylated tubulin or those of tyrosinated tubulin. Aβ42 was expressed in all neuron and retina with a combination of two GAL4 drivers, the pan-neuronal elav-GAL4 driver and pan-retinal gmr-GAL4 driver. No significant changes in the levels of acetyl tubulin or tyrosinated tubulin were detected in the Aβ42 fly brain. Two independent transgenic fly lines expressing Aβ42 at different expression levels (Aβ42#1 and Aβ42#2) yielded similar results. Genotypes: (control) elav-GAL4/Y;gmr-GAL4/+, (Aβ42#1) elav-GAL4/Y;gmr-GAL4/+;UAS-Aβ42/+ and (Aβ42#2) elav-GAL4/Y;gmr-GAL4/UAS-Aβ42.
(TIF)

S5 Fig RNAi-mediated knockdown of Sgg reduces tau phosphorylation at SP/TP sites. (A) Reduction in Sgg protein levels by the expression of Sgg RNAi in the retina. Heads lysates were subjected to western blotting with anti-GSK3 antibody. Mean ± SD, n = 5, *, *p <* 0.05, Student's t-test. Tubulin was used as loading control. Expression of UAS-SggRNAi was driven by the pan-retinal gmr-GAL4 driver. Note that Sgg RNAi is only expressed in the retina, while endogenous Sgg is ubiquitously expressed, and protein levels of Sgg were assessed by western blot of whole head lysate. Thus, the observed signal reflects not only Sgg protein in the retina, but also that in other cells in the head in which Sgg expression is not suppressed. Therefore, it is likely that reduction of Sgg protein in the retina is larger than the level shown here. Genotypes: (control) gmr-GAL4/+ and (Sgg RNAi) gmr-GAL4/+;UAS-Sgg RNAi/+. (B) RNAi-mediated knockdown of Sgg reduces tau phosphorylation at SP/TP sites. Western blots of fly heads expressing tau (tau) or that co-expressing tau and Sgg RNAi (tau+SggRNAi) with pan-tau antibody (tau46 and tauC) or antibodies that recognize phosphorylation status of tau at the SP/TP sites (pSer202, pThr231, PHF-1 and TAU-1). Tubulin was used as loading control. Mean ± SD, n = 5; *, *p* < 0.05, **, *p* < 0.01, ***, *p* < 0.005. Expression of tau and SggRNAi was driven by the pan-retinal gmr-GAL4 driver. Although residual Sgg-mediated phosphorylation of tau may be present, Sgg RNAi caused significant reduction in the levels of pSer202-tau, and pThr231-tau and PHF1 (24%, 15%, and 22% compared to control, respectively). Representative blots are shown. Genotypes: (tau) gmr-GAL4/+;UAS-tau/+ and (tau+Sgg RNAi) gmr-GAL4/+;UAS-Sgg RNAi/UAS-tau.
(TIF)

S6 Fig Expression of neither Aβ42 alone nor Aβ42 with Sgg RNAi causes a reduction in eye size. Heads of flies expressing the gmr-GAL4 driver alone (control), tau (tau), tau and Aβ42 (tau+Aβ42), or Aβ42 (Aβ42). The surface areas of the eyes are shown as mean ± SE (n = 6–8, one-way ANOVA, *p >* 0.05). Genotypes: (control) gmr-GAL4/+, (Sgg RNAi) gmr-GAL4/+;UAS-SggRNAi/+, (Aβ42+SggRNAi) gmr-GAL4/ UAS-Aβ42; UAS-SggRNAi/+ and (Aβ42) gmr-GAL4/UAS-Aβ42.
(TIF)

S7 Fig Knockdown of Par-1/MARK markedly decreases the levels of tau in the brain neurons. Western blots of fly heads expressing tau (tau) or that co-expressing tau and PAR-1 RNAi (tau+PAR-1RNAi) driven by elav-GeneSwitch with pan-tau antibody (tauC). Tubulin was used as loading control. Mean ± SD, n = 5; ***, *p*<0.005. Representative blots are shown. Genotypes are as follows: (tau) UAS-tau /+;elav-GeneSwitch/+ and (tau+PAR-1RNAi) UAS-tau /+;UAS-PAR1RNAi/elav-GeneSwitch. Transgene expression was induced by feeding newly eclosed flies RU486 for two days.
(TIF)

S8 Fig Phosphorylation levels of Sgg at Ser9 are increased by PAR-1 knockdown. Western blots of fly heads expressing gmr-GAL4 driver alone (control) or that expressing PAR-1 RNAi (PAR-1 RNAi) with anti-phospho-Ser21/9 antibody (pSer21/9) or a pan-GSK3 antibody (Sgg). Mean ± SD, n = 5; ***, *p*<0.005. Representative blots are shown. Genotypes: (control) gmr-GAL4/+ and (PAR-1RNAi) gmr-GAL4/+;UAS-PAR-1RNAi/+.
(TIF)

S9 Fig Phosphorylation levels of Sgg at Ser9 are not changed by expression of Aβ42. Aβ42 was expressed in all neuron and retina with a combination of two GAL4 drivers, the pan-neuronal elav-GAL4 driver and pan-retinal gmr-GAL4 driver. Western blots of fly heads expressing driver alone (control) or that expressing Aβ42 (Aβ42) with anti-phospho-Ser21/9 antibody (pSer21/9), or a pan-GSK3 antibody (Sgg). Mean ± SD, n = 5; *p*>0.05. Representative blots are shown. Genotypes: (control) elav-GAL4/Y;gmr-GAL4/+ and (Aβ42) elav-GAL4/Y;gmr-GAL4/UAS-Aβ42.
(TIF)

S10 Fig Tau phosphorylation at Ser238 is low in the retina of flies expressing tau alone or co-expressing tau and Aβ. (A). Western blotting of fly heads expressing tau alone or co-expressing tau and Aβ under the control of the pan-retinal gmr-GAL4 driver. No specific signal was detected with an anti-pSer238-tau antibody. (B) Western blotting of fly heads without expression of tau (control), expressing tau under the control of the pan-neuronal elav promoter (elav-tau (1) and elav-tau (2)), or expressing tau under the control of the gmr-GAL4 driver (gmr>tau). pSer238 was detected in elav-tau (1) and elav-tau (2), but not in gmr>tau. Genotypes: (control) gmr-GAL4/+, (tau) gmr-GAL4/+;UAS-tau/+, (tau+Aβ) gmr-GAL4/UAS-Aβ42;UAS-tau/+, (elav>tau(1)) elav-tau(1)/TM3Ser, (elav>tau(2)) elav-tau(2)/elav-tau(2), and (gmr>tau) gmr-GAL4/UAS-Aβ42;UAS-tau/+.
(TIF)

S1 Table Genotype of flies used in this study. (DOC)
