# Supplementary figures and images for: Stabilization of Microtubule-Unbound Tau via Tau Phosphorylation at Ser262/356 by Par-1/MARK Contributes to Augmentation of AD-Related Phosphorylation and Aβ42-Induced Tau Toxicity
Source: PLoS Genet. 2016 Mar 29;12(3):e1005917. doi: 10.1371/journal.pgen.1005917 (PMC4811436; doi:10.1371/journal.pgen.1005917)

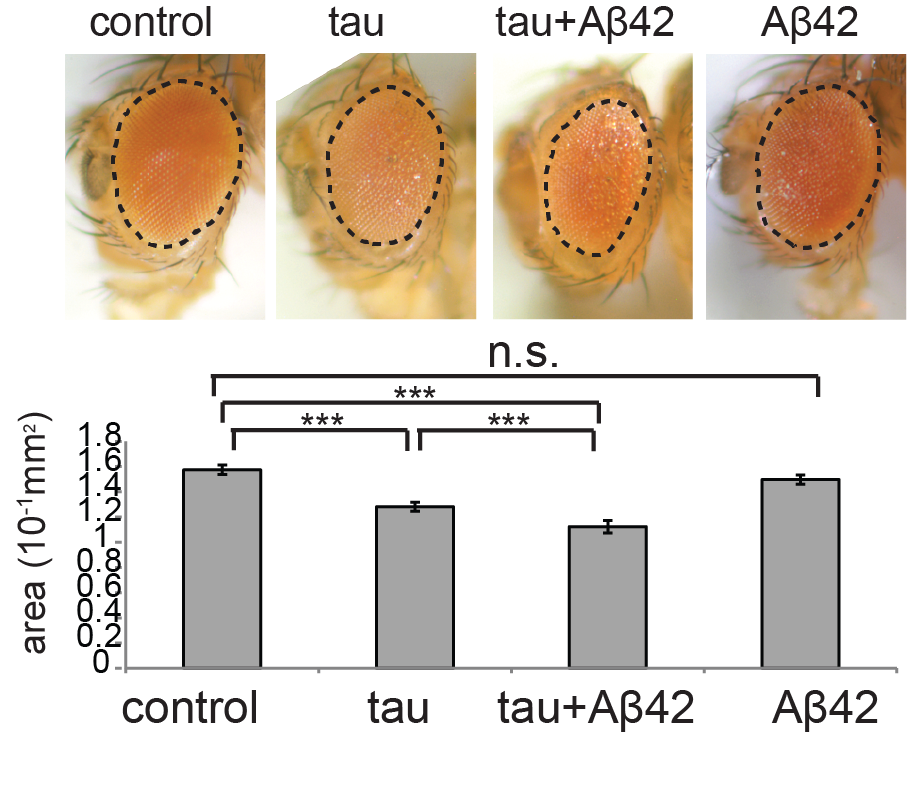

Supplement: S1 Fig — External eyes of flies expressing the gmr-gal4 driver alone (control), human tau (tau), tau and Aβ42 (tau+Aβ42) or Aβ42 (Aβ42). The surface areas of the external eyes are shown as mean ± SE (n = 6–8, one-way ANOVA, n = 5; ***, p<0.005, n.s., not significant (p>0.05)). Genotypes are as follows: (control) gmr-GAL4/+, (tau) gmr-GAL4/+;UAS-tau/+, (tau+Aβ42) gmr-GAL4/UAS-Aβ42;UAS-tau/+ and (Aβ42) gmr-GAL4/UAS-Aβ42. Transgene expression was driven by gmr-GAL4. (TIF) [file pgen.1005917.s001.tif]

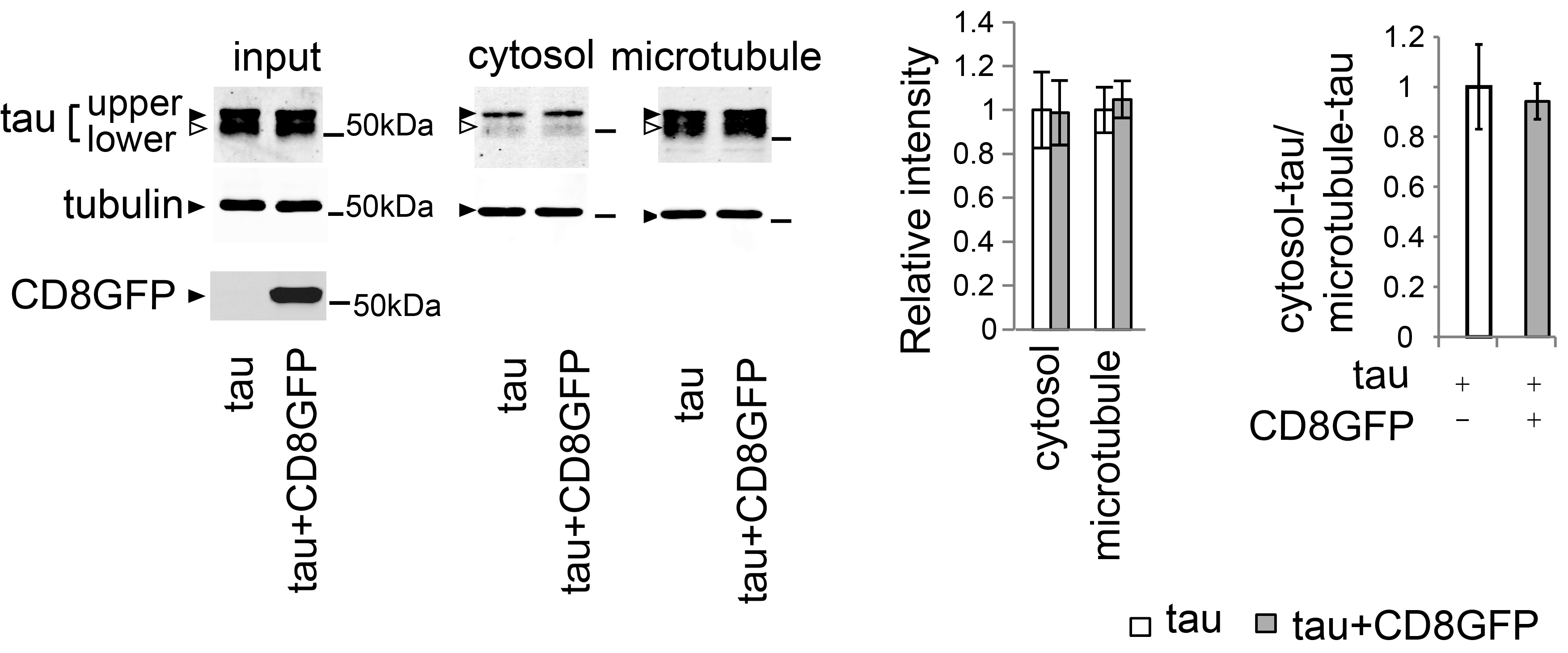

Supplement: S2 Fig — The levels of tau and tubulin in the lysate of fly heads expressing tau alone (tau) or co-expressing tau and non-toxic proteins in the secretory pathway CD8GFP (tau+CD8GFP) before sedimentation (input), in the supernatant (cytosol) and in the pellet containing microtubules (microtubule) were analyzed by western blotting by using anti-tau antibody. The same amount of proteins from each genotype was loaded. Expression of CD8GFP was confirmed by western blotting with anti-GFP antibody (CD8GFP). Mean ± SD, n = 5; p > 0.05 by Student's t-test. Representative blots are shown. Transgene expression was driven by gmr-GAL4. Genotypes are as follows: (tau) gmr-GAL4/+;UAS-tau/+ and (tau+CD8GFP) gmr-GAL4/UAS-CD8GFP;UAS-tau/+. (TIF) [file pgen.1005917.s002.tif]

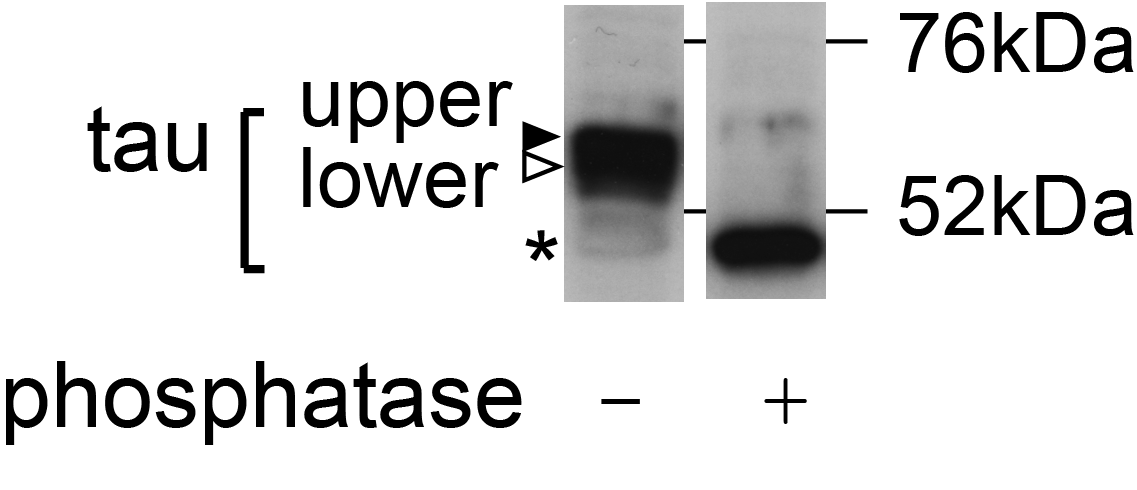

Supplement: S3 Fig — Western blots of lysate of fly heads expressing tau with or without phosphatase treatment with anti-tau antibody. Following phosphatase treatment, the two tau bands (indicated by arrowheads) merged and were detected as a single faster-migrating band (indicated by asterisk). Transgene expression was driven by gmr-GAL4. The fly genotype is gmr-GAL4/+;UAS-tau/+. (TIF) [file pgen.1005917.s003.tif]

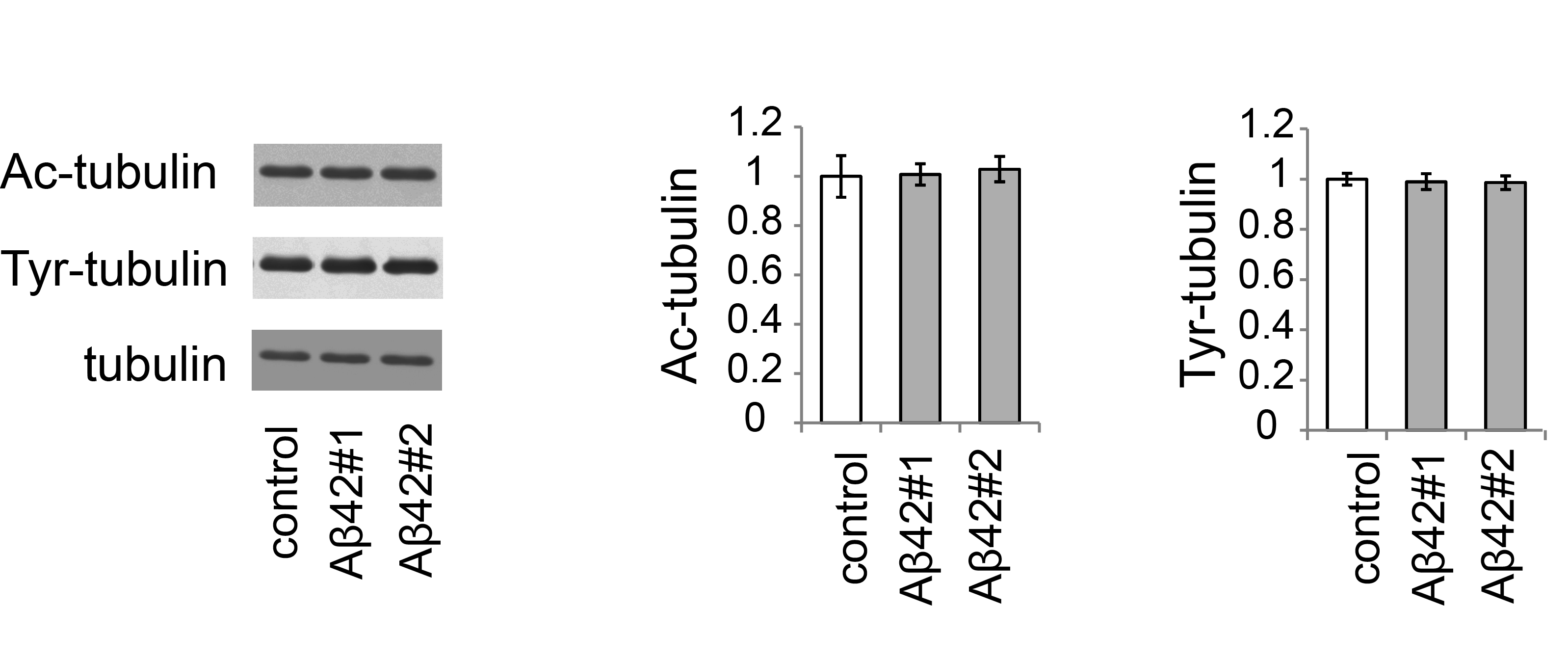

Supplement: S4 Fig — Aβ42 was expressed in all neuron and retina with a combination of two GAL4 drivers, the pan-neuronal elav-GAL4 driver and pan-retinal gmr-GAL4 driver. No significant changes in the levels of acetyl tubulin or tyrosinated tubulin were detected in the Aβ42 fly brain. Two independent transgenic fly lines expressing Aβ42 at different expression levels (Aβ42#1 and Aβ42#2) yielded similar results. Genotypes: (control) elav-GAL4/Y;gmr-GAL4/+, (Aβ42#1) elav-GAL4/Y;gmr-GAL4/+;UAS-Aβ42/+ and (Aβ42#2) elav-GAL4/Y;gmr-GAL4/UAS-Aβ42. (TIF) [file pgen.1005917.s004.tif]

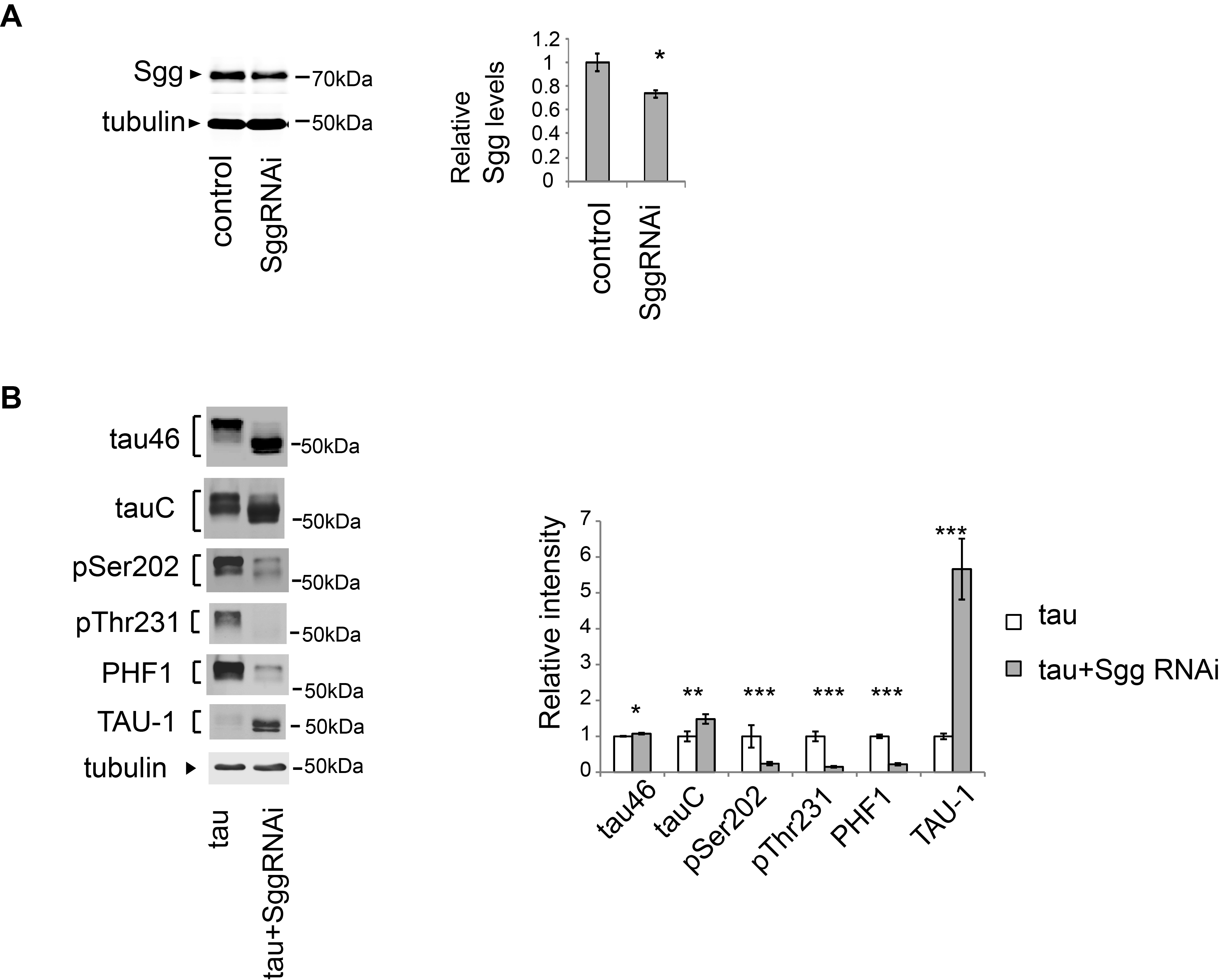

Supplement: S5 Fig — (A) Reduction in Sgg protein levels by the expression of Sgg RNAi in the retina. Heads lysates were subjected to western blotting with anti-GSK3 antibody. Mean ± SD, n = 5, *, p < 0.05, Student's t-test. Tubulin was used as loading control. Expression of UAS-SggRNAi was driven by the pan-retinal gmr-GAL4 driver. Note that Sgg RNAi is only expressed in the retina, while endogenous Sgg is ubiquitously expressed, and protein levels of Sgg were assessed by western blot of whole head lysate. Thus, the observed signal reflects not only Sgg protein in the retina, but also that in other cells in the head in which Sgg expression is not suppressed. Therefore, it is likely that reduction of Sgg protein in the retina is larger than the level shown here. Genotypes: (control) gmr-GAL4/+ and (Sgg RNAi) gmr-GAL4/+;UAS-Sgg RNAi/+. (B) RNAi-mediated knockdown of Sgg reduces tau phosphorylation at SP/TP sites. Western blots of fly heads expressing tau (tau) or that co-expressing tau and Sgg RNAi (tau+SggRNAi) with pan-tau antibody (tau46 and tauC) or antibodies that recognize phosphorylation status of tau at the SP/TP sites (pSer202, pThr231, PHF-1 and TAU-1). Tubulin was used as loading control. Mean ± SD, n = 5; *, p < 0.05, **, p < 0.01, ***, p < 0.005. Expression of tau and SggRNAi was driven by the pan-retinal gmr-GAL4 driver. Although residual Sgg-mediated phosphorylation of tau may be present, Sgg RNAi caused significant reduction in the levels of pSer202-tau, and pThr231-tau and PHF1 (24%, 15%, and 22% compared to control, respectively). Representative blots are shown. Genotypes: (tau) gmr-GAL4/+;UAS-tau/+ and (tau+Sgg RNAi) gmr-GAL4/+;UAS-Sgg RNAi/UAS-tau. (TIF) [file pgen.1005917.s005.tif]

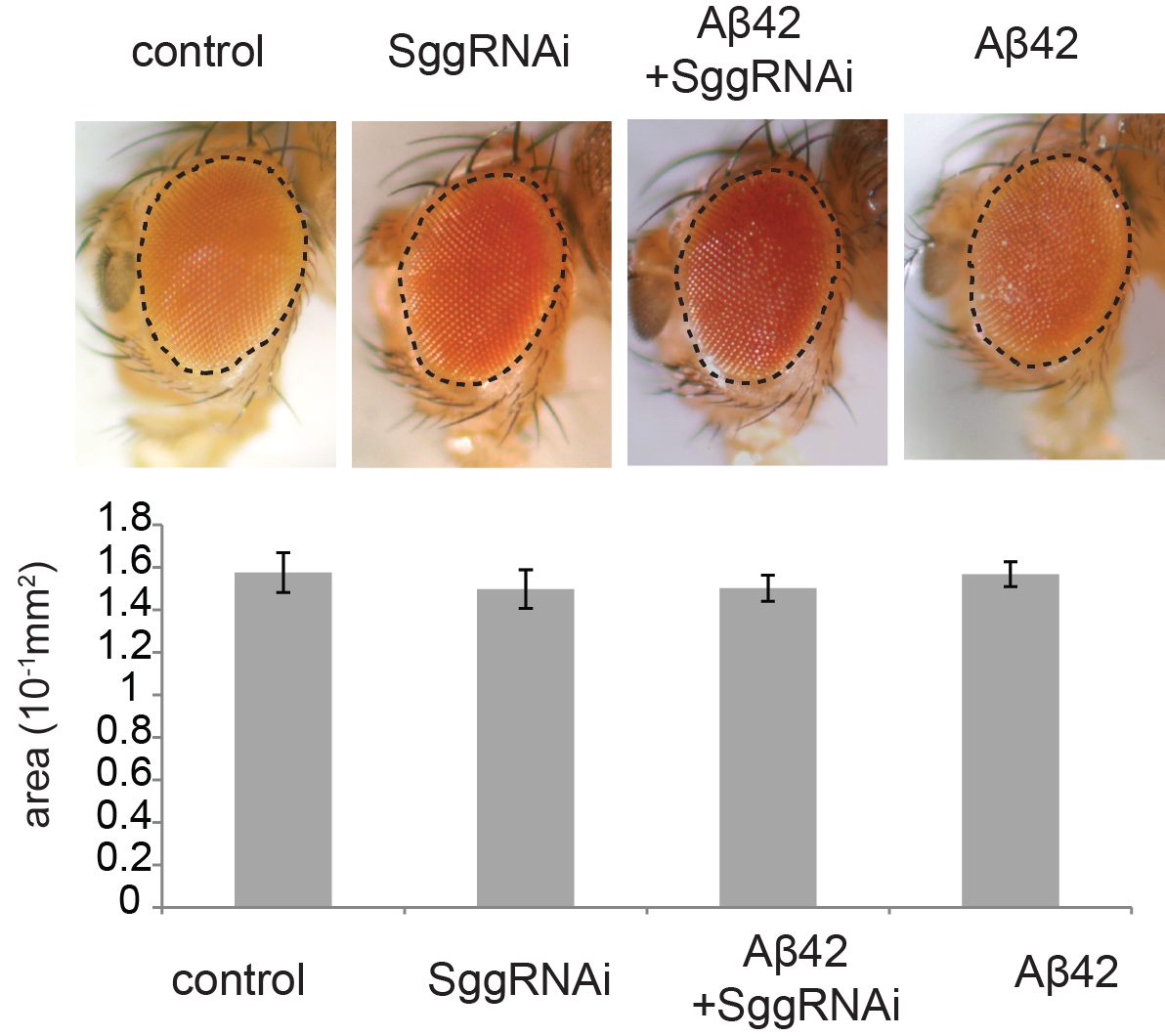

Supplement: S6 Fig — Heads of flies expressing the gmr-GAL4 driver alone (control), tau (tau), tau and Aβ42 (tau+Aβ42), or Aβ42 (Aβ42). The surface areas of the eyes are shown as mean ± SE (n = 6–8, one-way ANOVA, p > 0.05). Genotypes: (control) gmr-GAL4/+, (Sgg RNAi) gmr-GAL4/+;UAS-SggRNAi/+, (Aβ42+SggRNAi) gmr-GAL4/ UAS-Aβ42; UAS-SggRNAi/+ and (Aβ42) gmr-GAL4/UAS-Aβ42. (TIF) [file pgen.1005917.s006.tif]

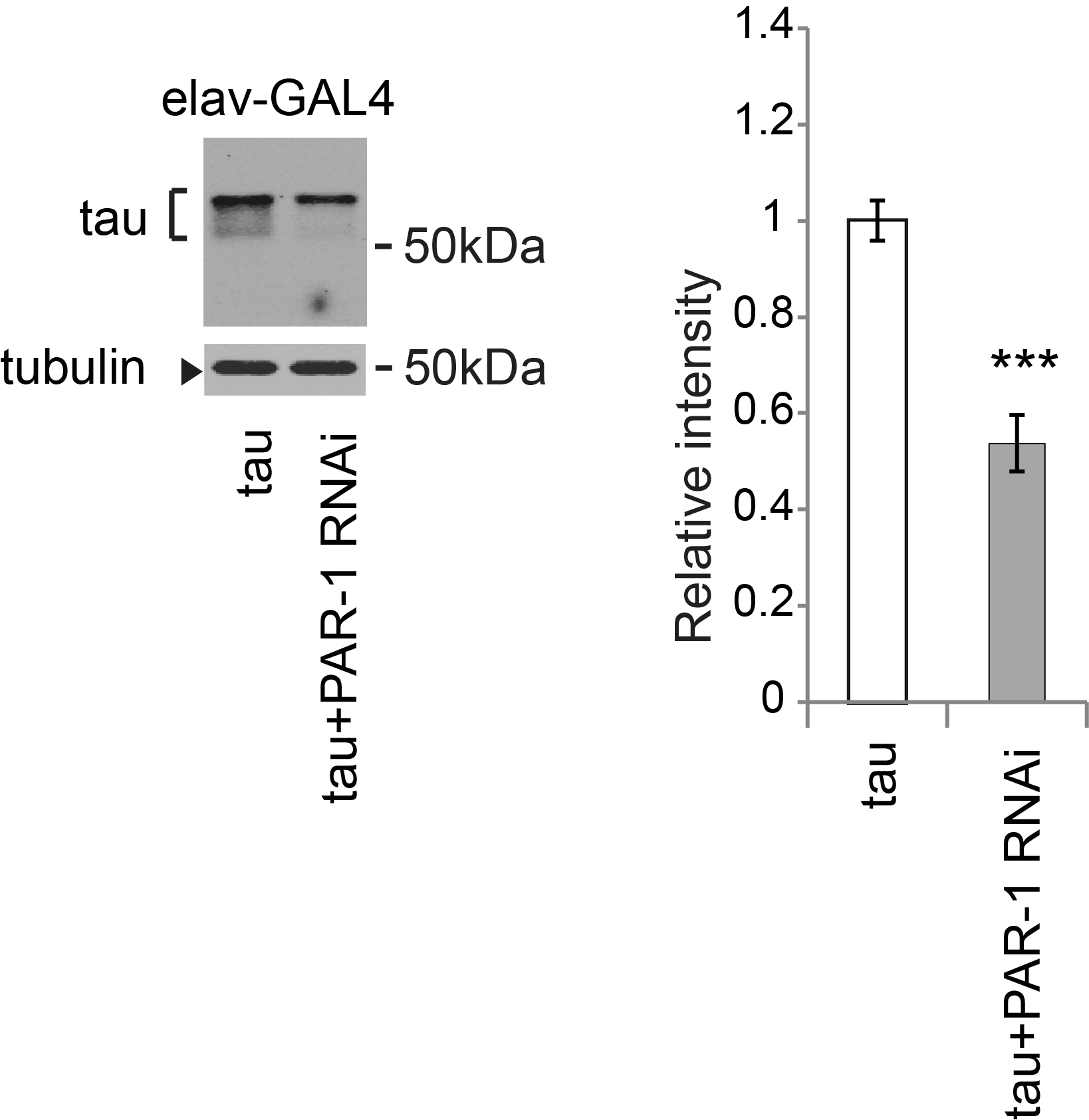

Supplement: S7 Fig — Western blots of fly heads expressing tau (tau) or that co-expressing tau and PAR-1 RNAi (tau+PAR-1RNAi) driven by elav-GeneSwitch with pan-tau antibody (tauC). Tubulin was used as loading control. Mean ± SD, n = 5; ***, p<0.005. Representative blots are shown. Genotypes are as follows: (tau) UAS-tau /+;elav-GeneSwitch/+ and (tau+PAR-1RNAi) UAS-tau /+;UAS-PAR1RNAi/elav-GeneSwitch. Transgene expression was induced by feeding newly eclosed flies RU486 for two days. (TIF) [file pgen.1005917.s007.tif]

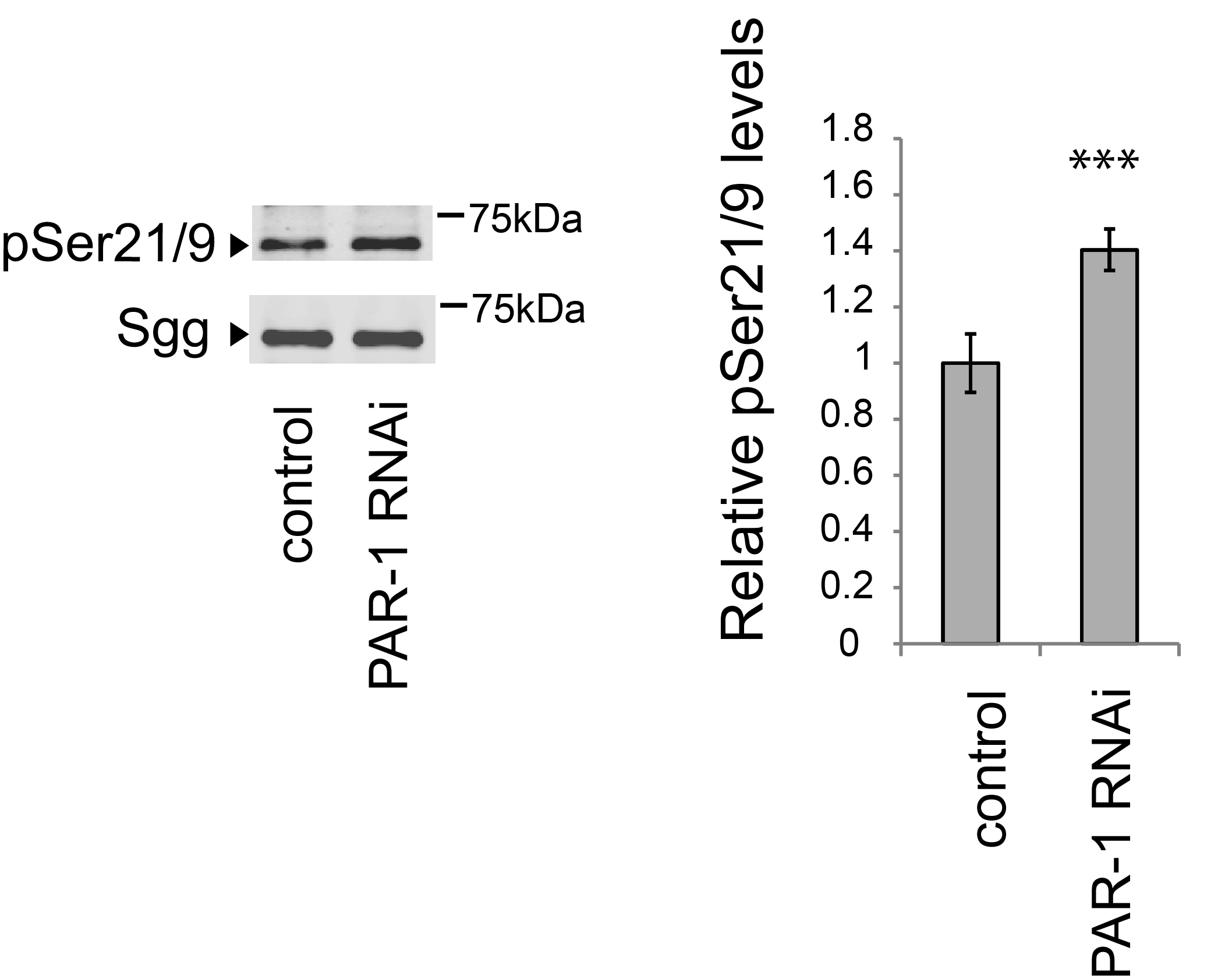

Supplement: S8 Fig — Western blots of fly heads expressing gmr-GAL4 driver alone (control) or that expressing PAR-1 RNAi (PAR-1 RNAi) with anti-phospho-Ser21/9 antibody (pSer21/9) or a pan-GSK3 antibody (Sgg). Mean ± SD, n = 5; ***, p<0.005. Representative blots are shown. Genotypes: (control) gmr-GAL4/+ and (PAR-1RNAi) gmr-GAL4/+;UAS-PAR-1RNAi/+. (TIF) [file pgen.1005917.s008.tif]

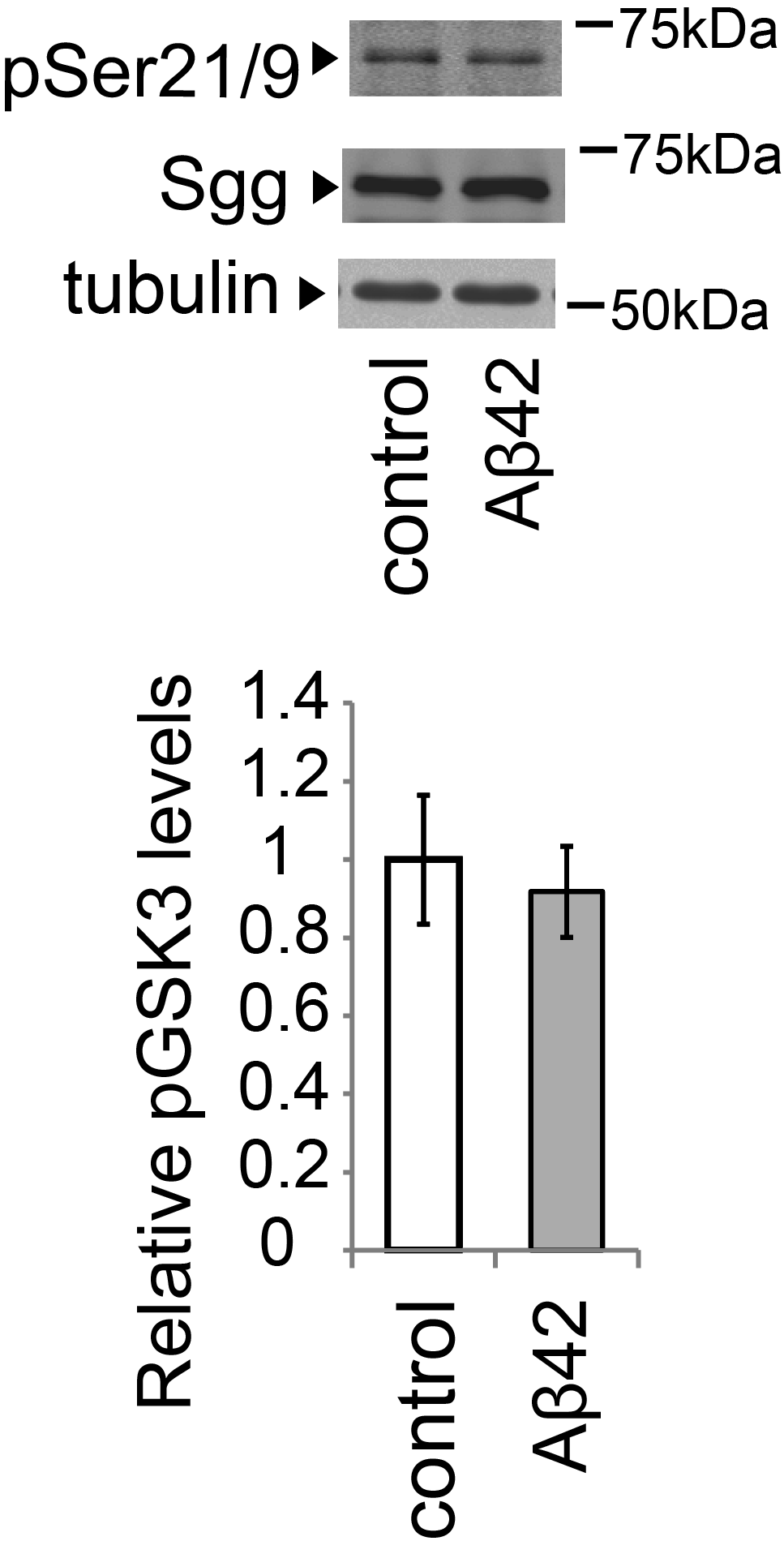

Supplement: S9 Fig — Aβ42 was expressed in all neuron and retina with a combination of two GAL4 drivers, the pan-neuronal elav-GAL4 driver and pan-retinal gmr-GAL4 driver. Western blots of fly heads expressing driver alone (control) or that expressing Aβ42 (Aβ42) with anti-phospho-Ser21/9 antibody (pSer21/9), or a pan-GSK3 antibody (Sgg). Mean ± SD, n = 5; p>0.05. Representative blots are shown. Genotypes: (control) elav-GAL4/Y;gmr-GAL4/+ and (Aβ42) elav-GAL4/Y;gmr-GAL4/UAS-Aβ42. (TIF) [file pgen.1005917.s009.tif]

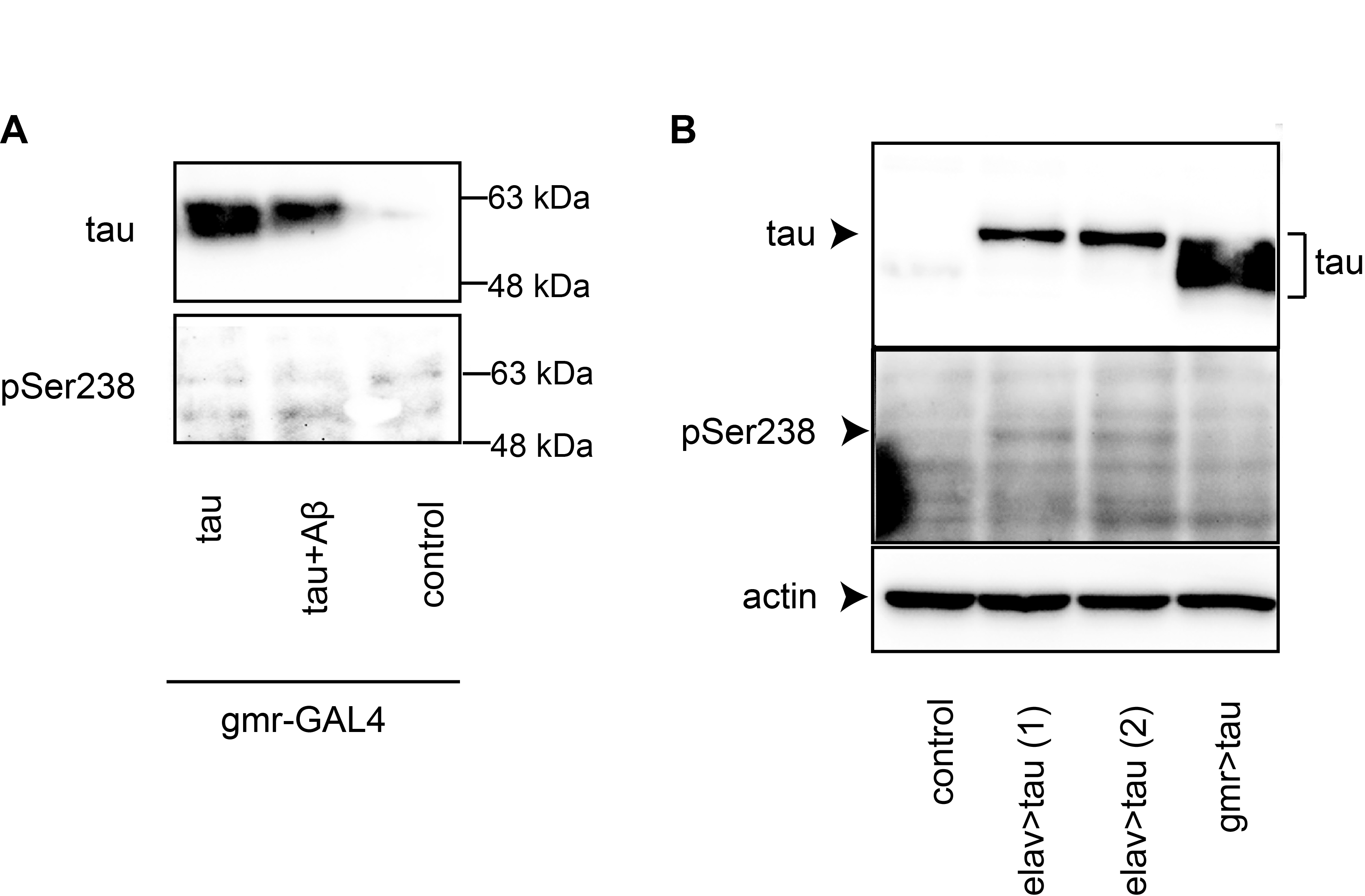

Supplement: S10 Fig — (A). Western blotting of fly heads expressing tau alone or co-expressing tau and Aβ under the control of the pan-retinal gmr-GAL4 driver. No specific signal was detected with an anti-pSer238-tau antibody. (B) Western blotting of fly heads without expression of tau (control), expressing tau under the control of the pan-neuronal elav promoter (elav-tau (1) and elav-tau (2)), or expressing tau under the control of the gmr-GAL4 driver (gmr>tau). pSer238 was detected in elav-tau (1) and elav-tau (2), but not in gmr>tau. Genotypes: (control) gmr-GAL4/+, (tau) gmr-GAL4/+;UAS-tau/+, (tau+Aβ) gmr-GAL4/UAS-Aβ42;UAS-tau/+, (elav>tau(1)) elav-tau(1)/TM3Ser, (elav>tau(2)) elav-tau(2)/elav-tau(2), and (gmr>tau) gmr-GAL4/UAS-Aβ42;UAS-tau/+. (TIF) [file pgen.1005917.s010.tif]
