# Supplementary material for: Stabilization of Microtubule-Unbound Tau via Tau Phosphorylation at Ser262/356 by Par-1/MARK Contributes to Augmentation of AD-Related Phosphorylation and Aβ42-Induced Tau Toxicity
Source: PLoS Genet. 2016 Mar 29;12(3):e1005917. doi: 10.1371/journal.pgen.1005917 (PMC4811436; doi:10.1371/journal.pgen.1005917)
Supplement: S1 Table — (DOC) [file pgen.1005917.s011.doc]

| Figure 1 | tau | +/+;gmr-GAL4/+;UAS-tau/+ |
| --- | --- | --- |
|  | tau+Aβ42 | +/+;UAS-Aβ42/gmr-GAL4;UAS-tau/+ |
|  | tau+Aβ42#1 | +/+;gmr-GAL4/+;UAS-tau/UAS-Aβ42 |
|  | tau+Aβ42#2 | +/+;UAS-Aβ42/gmr-GAL4;UAS-tau/+ |
| Figure 2 | tau | +/+;gmr-GAL4/+;UAS-tau/+ |
|  | tau+SggRNAi | +/+;gmr-GAL4/+;UAS-tau/UAS-SggRNAi |
|  | tau+Aβ42 | +/+;UAS-Aβ42/gmr-GAL4;UAS-tau/+ |
|  | SggRNAi+tau | +/+;gmr-GAL4/+;UAS-tau/UAS-SggRNAi |
|  | SggRNAi+tau+Aβ42 | +/+;UAS-Aβ42/gmr-GAL4;UAS-tau/UAS-SggRNAi |
|  | SggRNAi | +/+;gmr-GAL4/+;UAS-SggRNAi/+ |
|  | SggRNAi+Aβ42 | +/+;UAS-Aβ42/gmr-GAL4;UAS-tau/UAS-SggRNAi |
| Figure 3 | S2A | +/+;gmr-GAL4/+;UAS-S2Atau/+ |
|  | S2A+Aβ42 | +/+;UAS-Aβ42/gmr-GAL4;UAS-S2Atau/+ |
|  | tau+Aβ42 | +/+;UAS-Aβ42/gmr-GAL4;UAS-tau/+ |
| Figure 4 | tau | +/+;gmr-GAL4/+;UAS-tau/+ |
|  | tau+PAR-1RNAi | +/+;gmr-GAL4/+;UAS-tau/UAS-PAR-1RNAi |
|  | tau+PAR-1OE | +/+;gmr-GAL4/+;UAS-tau/UAS-PAR-1 |
|  | S2A | +/+;gmr-GAL4/+;UAS-S2Atau/+ |
|  | S2A+PAR-1RNAi | +/+;gmr-GAL4/+;UAS-S2Atau/UAS-PAR-1RNAi |
|  | S2A+PAR-1OE | +/+;gmr-GAL4/+;UAS-S2Atau/UAS-PAR-1 |
| Figure 5 | tau | +/+;gmr-GAL4/+;UAS-tau/+ |
|  | tau+Aβ42 | +/+;UAS-Aβ42/gmr-GAL4;UAS-tau/+ |
|  | PAR-1RNAi+tau | +/+;gmr-GAL4/+;UAS-tau/UAS-PAR-1RNAi |
|  | PAR-1RNAi+tau+Aβ42 | +/+;UAS-Aβ42/gmr-GAL4;UAS-tau/UAS-PAR-1RNAi |
|  | Aβ42+tau | +/+;UAS-Aβ42/gmr-GAL4;UAS-tau/+ |
|  | Aβ42+tau+PAR-1RNAi | +/+;UAS-Aβ42/gmr-GAL4;UAS-tau/UAS-PAR-1RNAi |
| Figure 6 | Aβ42+tau | +/+;UAS-Aβ42/gmr-GAL4;UAS-tau/+ |
|  | Aβ42+tau+SggRNAi | +/+;UAS-Aβ42/gmr-GAL4;UAS-tau/UAS-SggRNAi |
| Figure 7 | S2A | +/+;gmr-GAL4/+;UAS-S2Atau/+ |
|  | S2A+Aβ42 | +/+;UAS-Aβ42/gmr-GAL4;UAS-S2Atau/+ |
|  | PAR-1RNAi+tau | +/+;gmr-GAL4/+;UAS-tau/UAS-PAR-1RNAi |
|  | PAR-1RNAi+tau+Aβ42 | +/+;UAS-Aβ42/gmr-GAL4;UAS-tau/UAS-PAR-1RNAi |
|  | tau+PAR-1OE | +/+;gmr-GAL4/+;UAS-tau/UAS-PAR-1 |

**Table S1. Fly genotypes.**
